# Exosomal Delivery Enhances the Antiproliferative Effects of Acid-Hydrolyzed *Apiaceae* Spice Extracts in Breast Cancer Cells

**DOI:** 10.3390/foods13172811

**Published:** 2024-09-04

**Authors:** Jared L. Scott, Ramesh C. Gupta, Farrukh Aqil, Jeyaprakash Jeyabalan, David J. Schultz

**Affiliations:** 1 Department of Biology, University of Louisville, Louisville, KY 40292, USA; jared.scott@louisville.edu; 2 Brown Cancer Center and Department of Pharmacology and Toxicology, University of Louisville, Louisville, KY 40202, USA; ramesh.gupta@louisville.edu; 3 Brown Cancer Center and Department of Medicine, University of Louisville, Louisville, KY 40202, USA; farrukh.aqil@louisville.edu; 4 3P Biotechnologies, Inc., Louisville, KY 40202, USA; jp3pbiotech@gmail.com

**Keywords:** phytochemistry, antiproliferative activity, *Apiaceae* spices, breast cancer, colostrum exosomes, drug delivery

## Abstract

Breast cancer remains a leading cause of death worldwide. The *Apiaceae* plant family includes many culinary spices that have been shown to have medicinal properties. Many phytochemicals exhibit potent bioactivities but often suffer from poor uptake and oral bioavailability. Bovine milk and colostrum exosomes are a compelling drug delivery platform that could address this issue; these natural nanoparticles can be loaded with hydrophilic and lipophilic small molecules and biologics, resulting in lower doses needed to inhibit cancer growth. Ethanolic extracts of eight *Apiaceae* spices were examined for phytochemical content and antiproliferative potential. Acid hydrolysis (AH) was employed to remove glycosides, asses its impacts on extract efficacy, and evaluate its effects on exosome loading and subsequent formulation efficacy. Antiproliferative activity was assessed through MTT assays on T-47D, MDA-MB-231, and BT-474 breast cancer cells; all extracts exhibited broad antiproliferative activity. AH enhanced the bioactivity of cumin, caraway, and fennel in T-47D cells. Celery, cumin, anise, and ajwain showed the highest activity and were assayed in exosomal formulations, which resulted in reduced doses required to inhibit cellular proliferation for all extracts except AH-cumin. *Apiaceae* spice extracts demonstrated antiproliferative activities that can be improved with AH and further enhanced with exosomal delivery.

## 1. Introduction

Breast cancer is the most common type of cancer in women, with 2.1 million diagnosed in 2018 according to the 2020 world cancer report, and the American Cancer Society has estimated that there would be over 300 thousand new diagnoses in the US alone in 2023 [1,2]. Breast cancer kills more women in the US than any other disease except heart disease according to the 2020 National Vital Statistics Report by the US CDC [3]. Breast cancer is often treated with mastectomies paired with chemotherapy and radiation treatments. However, these conventional treatments often come with significant and detrimental side effects. Additionally, mastectomies do not guarantee breast cancer prevention and are both painful and disfiguring [4,5]. The adverse effects associated with chemotherapy and radiation can lead some patients to opt not to finish treatment or to refuse chemotherapy all together [5]. Thus, approaches that maintain efficacy while lowering side effects are of great interest. Natural plant extracts exhibit antiproliferative and chemopreventive effects and have lower toxicity when compared to modern chemotherapeutics, and thus have interesting potential as breast cancer treatments [6,7,8].

One source of potential anticancer plant compounds is the *Apiaceae* plant family, more commonly known as the carrot or parsley family, which is found in human diets worldwide. Beyond carrots, fennel, celery, and many other vegetables, this family is also known for several aromatic herbs and spices such as coriander/cilantro, caraway, dill, anise, and cumin. Scientific studies have revealed that many *Apiaceae* plants produce a rich array of phytochemicals, including alkaloids, terpenoids, and phenolics. These compounds exhibit antioxidant, chemopreventive, and anticancer bioactivities [8,9,10,11]. Unfortunately, plant extracts often face challenges such as poor uptake and low bioavailability, resulting in higher concentrations of extracts required to produce bioactive effects in vivo. To overcome these limitations, various scientific studies are focused on enhancing bioavailability through methods like nanoparticle delivery [12,13,14]. One method for reducing the required dose for effective chemotherapy treatments is the encapsulation of anticancer chemicals in nanoparticles, and a class of membrane-bound vesicles, known as exosomes, represents an interesting option.

Exosomes are naturally occurring nanoparticles involved in cell-to-cell communication and can transport proteins, nucleic acids, lipids, and small molecules directly into a target cell’s cytosol [15]. The bodily fluids of multicellular organisms such as saliva, sweat, spinal fluid, hemolymph, blood, and milk contain exosomes and thus are potential sources of exosomes that could be harvested and used for nanoparticle drug delivery applications. Cancer cells also produce exosomes, carry factors that enhance cell survival, affect the ability to invade surrounding tissues, and enhance growth and cellular proliferation by increasing angiogenesis. Specific RNAs carried by exosomes are now used to screen for some cancers [16,17,18]. Many exosome sources for drug delivery, such as human cell cultures, bring risks of introducing these cellular effectors along with drug formulations targeted for delivery. Milk exosomes are a promising platform for drug delivery as these nanoparticles can be continually harvested without sacrificing the organism, are already a part of the human diet in dairy products, and are similar to exosomes found in human breast milk. Further, milk exosomes survive the digestive process and are absorbed by intestinal cells [19,20,21]. In addition to providing a scalable source of materials that is already in the human diet, milk exosomes are a flexible nanoparticle platform for the delivery of exogenous target molecules. Milk exosomes have been synthetically loaded and used to deliver small molecules and nucleic acids into cancer cells, yielding promising results [21]. Recently, plant extracts of black bean (*Phaseolus vulgaris*) have been loaded into exosomes derived from cell cultures of several different types of cancer (MCF-7: breast, PC3: prostate, Caco2: colon, and HepG2: liver) and they have been shown to have increased antiproliferative effects in comparison to the free extracts when administered back to their source cancers [22]. If plant extracts that exhibit antiproliferative effects can overcome the pro-cancer factors inherent in these cancer exosomes, loading plant extracts into milk exosomes may further increase their impact. Additionally, by lowering the concentration of bioactive agents needed to inhibit the growth of cancer cells, the amount of bioactive agents that healthy cells are exposed to is also lowered, potentially reducing the side effects felt by the patient.

In addition to poor bioavailability, plants often store specialized metabolites in glycosylated forms that modulate toxic bioactivities for targeted release in response to certain stimuli such as herbivory or infection [23,24]. Glycosylated forms of phytochemicals are typically more polar due to the addition of one or more sugar moieties. Polar molecules have lower efficiency of exosome loading compared with non-polar molecules, and since many phytochemicals found in typical plant extracts tend to be glycosylated, the efficiency of exosome loading can be limited [25]. Additionally, we have shown that human lung cancer A549 cells treated with a mixture of anthocyanidins (aglycones) showed higher growth inhibition compared to their respective glycosides (anthocyanins) [26]. Acid hydrolysis (AH) is a non-enzymatic method for the deglycosylation of phytochemicals and thus the influence of AH on *Apiaceae* spice extracts was tested on antiproliferative bioactivity and loading potential in bovine milk exosomes. Several *Apiaceae* spices are closely related and tend to share similar bioactive profiles with minor variations in phytochemical decorations, such as the location of hydroxyl groups, double bonds, and/or methyl groups, and relative quantitative ratios of phytochemicals produced.

This exploratory study seeks to determine the impact of acid hydrolysis and exosomal delivery on the antiproliferative effects of *Apiaceae* spice extracts. Additionally, this study seeks to determine a hierarchical ranking of the antiproliferative effects of eight common *Apiaceae* spices when acid hydrolysis and exosomal delivery are utilized. We hypothesize that several seed extracts of these closely related plant species will exhibit antiproliferative effects in breast cancer cell models and this activity could be improved through acid hydrolysis and exosomal delivery. This is the first study to combine the acid hydrolysis of plant extracts with exosomal loading and delivery. Additionally, this is the first study to describe the loading of whole plant extracts into bovine colostrum exosomes and how that may be improved with acid hydrolysis.

## 2. Materials and Methods

### 2.1. Phytochemical Analysis

**Figure 1:**
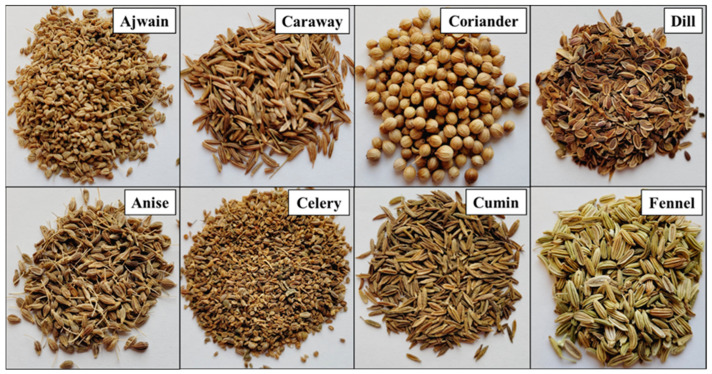
*Apiaceae* spice seeds ajwain, caraway, coriander, dill, anise, celery, cumin, fennel.

Organically grown *Apiaceae* seeds (Figure 1) of ajwain (*Trachyspermum ammi* sourced from India), anise (*Pimpinella anisum* sourced from Egypt), caraway (*Carum carvi* sourced from Egypt), celery (*Apium graveolens* sourced from Egypt), coriander (*Coriandrum sativum* sourced from Egypt), cumin (*Cuminum cyminum* sourced from Turkey), dill (*Anethum graveolens* sourced from India), and fennel (*Foeniculum vulgare* sourced from Turkey) were purchased from Mountain Rose Herbs (Eugene, OR, USA).

#### 2.1.1. Extract Preparations

*Apiaceae* spice seeds were ground to a homogenous fine powder using a Mr. Coffee IDS77 Blade Grinder (Mr. Coffee, Cleveland, OH, USA). All extractions were conducted using a ratio of 100g ground seeds to 400 mL solvent. After each sonication, the insoluble materials were removed by centrifugation at 4500× *g* for 10min at 20 °C, and then the supernatant was collected and filtered through a 0.44 µm cellulose acetate/cellulose nitrate membrane filter (Millipore Sigma, Burlington, MA, USA) under vacuum. Each of the three filtered extractions of the seed material was combined, separately for each spice, frozen at −80 °C, and then freeze-dried in a 4.5 L FreeZone −84 °C freeze dryer (Labconco, Kanas City, MO, USA). The dried extracts were collected and stored at −20 °C.

#### 2.1.2. Total Phenolics, Terpenoids, and Alkaloids

Spice extracts (100 mg each) were resuspended in 1 mL of either 80% ethanol for terpenoid and phenolic analysis or in 1.2 N HCl for alkaloid analysis. Total terpenoid content was measured against a standard curve of linalool ((±)-3,7-dimethyl-1,6-octadien-3-ol, Fisher Scientific, Pittsburgh, PA, USA) as described in [27]. The total phenolics were determined by a modified method [28,29,30], using gallic acid as a standard. The total alkaloid content was measured using a modified method [31,32], which utilizes atropine (tropine DL-tropate 99% from Fisher Scientific, Pittsburgh, PA, USA) as a standard. The results were measured using a SpectraMax M2 UV/Vis microplate reader (Molecular Devices, San Jose, CA, USA) or a GENESYS™ 10 UV-Vis Spectrophotometer (Thermo Fisher Scientific, Waltham, MA, USA) for alkaloids.

#### 2.1.3. Phytochemical Analysis by HPLC

To further examine and compare the complex mixtures of phenolic compounds present in each extract, high-performance liquid chromatography (HPLC) analysis was conducted, utilizing an adapted protocol [29,33]. Reverse phase separation was carried out at 30 °C on a 250 mm × 4.6 mm HiChrom Alltima HP 5 µm C18-AQ HPLC column (Avantor, Radnor, PA, USA) fitted with an Alltima 5 µm AQ column guard filter using two mobile phases of 1% acetic acid (mobile phase A) in water and 1% acetic acid in methanol (mobile phase B). The method consisted of a gradient from 10% to 50% solvent B over 5 min and then 50% to 100% solvent B over 15 min, with a flow of 1.25 mL min^−^^1^. After each run the column was washed with 100% B for 5 min before being returned to 10% B and allowed to equilibrate for 5 min before the next sample was loaded; this solvent gradient is displayed on the HPLC chromatographs to illustrate the decreasing polarity of the compounds as they are eluted from the column. The injection volume was 20 µL of extract (10 mg/mL), and all measurements were repeated in triplicate. An HP 1100 HPLC (Agilent Technologies, Santa Clara, CA, USA) equipped with a UV/Vis diode-array detector (DAD) was set to record with a response time of 1 s at 280 nm with a bandwidth of 16nm, using a 500 nm reference wavelength with a bandwidth of 100 nm. All solvents were HPLC grade (Avantor, Radnor, PA, USA).

### 2.2. Acid Hydrolysis

The acid hydrolysis of the spice extracts was performed using a modified method [33]. Briefly, 10 mg/mL of dry *Apiaceae* spice extract was incubated with a hydrolysis mixture of 1.2 N HCl in 50% methanol for 4 h, and the extracts were then neutralized with NaOH. The extracts were dried in a Centrivap Concentrator (Labconco, Kanas City, MO, USA) until completely dry, typically for 24 h. To reduce salt content, the extracts were reconstituted in 100% methanol, sonicated for 10 min, and centrifuged at 4500× *g* for 5 min. The supernatant was transferred to a screw cap glass vial and again dried completely in a Centrivap. This extract was then reconstituted using 80% ethanol and filter sterilized with a 0.2 µm PTFE filter and stored at −20 °C until needed for exosome loading. A portion of the hydrolyzed extract was used for exosome loading and the remaining extract volume was measured, dried once more, and reconstituted at 1/10th the measured volume to produce a 10× concentrated extract for cell culture assays. Additionally, to control for the impact of the AH process, “mock” AH extracts were subjected to the AH process as described but without the addition of acid and are further referred to as “processed extracts”.

### 2.3. Exosomal Formulations of Apiaceae Spices

#### 2.3.1. Exosomes

Bovine milk exosomes, isolated and enriched, were kindly donated by 3P Biotechnologies, Inc. (Louisville, KY, USA). Their industrial-scale proprietary method involves both ultracentrifugation and ultrafiltration systems to yield naturally produced and highly purified exosome nanoparticles derived from bovine milk. These exosomes had an average diameter of 154.0 ± 5.6 nm, zeta potential of −7.16 ± 0.27 mV, and a PDI of 0.287 ± 0.007.

#### 2.3.2. Drug Loading

An exosome drug loading protocol was adapted from previous reports [21]. Briefly, 1 mL of ethanolic spice extracts (10 mg/mL processed or AH) was gradually added and mixed with exosomes (20 mg exosomal protein) suspended in PBS in a beaker with a stir bar set to low speed and incubated for 30 min at room temperature, followed by centrifugation at 10,000× *g* to remove any unbound precipitants. The drug-loaded exosomes then underwent ultrafiltration using a Vivaspin^®^ 300 k molecular weight cutoff filter (Sartorius AG, Göttingen, Germany) to separate the exosomal formulation from unbound phytochemicals. The exosomal formulation was washed three times with PBS and then resuspended in PBS and stored at −80 °C until used. For cell culture, exosomal formulations were lyophilized and stored at −20 °C.

Exosomal formulations of processed and AH-*Apiaceae* seed extracts were characterized in terms of size, charge, and polydispersity index (PDI) with a Nano ZS Zetasizer (Malvern Instruments Limited, Malvern, UK). Particle counts were assayed with nanoparticle tracking analysis using a Particle Metrix ZetaView^®^ (Particle Metrix GmbH, Inning am Ammersee, Germany) according to the manufacturer’s instructions.

### 2.4. Assessment of Extract Loading

Extract loading was determined by analyzing the extract and exosome concentrations using HPLC and a standard BCA protein estimation kit, respectively, as described [21]. HPLC-DAD was used to measure the amount of extract in exosomal formulations. Briefly, 250 µL of the exosome formulations was added to 2.5 mL ice-cold ethanol and kept for 30 min at −80 °C. The reaction mixture was then centrifuged (10,000× *g* for 10 min) to separate the pellet. The supernatant was collected separated and dried in a Centrivap. The dried extract was reconstituted in 250 µL of 80% ethanol, filtered with a 0.2 µm PTFE filter, and transferred to an HPLC vial for immediate analysis as described above. The protein pellet was suspended in PBS and its concentration was determined by BCA.

### 2.5. Cancer Cell Growth Conditions

Three breast cancer cell lines, T-47D (HTB-133), MDA-MB-231 (HTB-26), and BT-474 (HTB-20), were obtained from the American Type Culture Collection (ATCC, Manassas, VA, USA). T-47D and BT-474 were grown in RPMI-1640 medium supplemented with 10% FBS, 2% Penn-Strep (Fisher Scientific, Pittsburgh, PA, USA), and 0.2 units/mL bovine insulin (Millipore Sigma, Burlington, MA, USA) at 37 °C with 5% CO_2_ supplied, while MDA-MB-231 cells were grown in Leibovitz’s L-15 medium (Fisher Scientific, Pittsburgh, PA, USA) with 10% FBS and 2% Penn-Strep at 37 °C with no CO_2_ supplied (this cell line does not require CO_2_ supplementation per ATCC’s instructions). The cells were grown with regular media changes every 2–3 days until they were 80% confluent in a T-75 cm^2^ flask (Avantor, Radnor, PA, USA). These three cell models represent the three most common breast cancer molecular subtypes (luminal subtype A: T-47D, luminal subtype B: BT-474, and triple negative/basal: MDA-MB-231).

### 2.6. MTT Cell Proliferation Assay

Cellular proliferation was measured using an MTT assay as described in [21]. BC T-47D and BT-474 cells were seeded at 5000 cells per well and MDA-MB-231 was seeded at 10,000 cells per well, in a 96-well plate except for two wells to serve as plate blanks. Cells were allowed to adhere overnight and treated with the initial *Apiaceae* seed extracts (In-E), processed extracts (Pro-E), and AH-extracts (AH-E) (0–1 mg/mL extract dry mass), or equivalent volumes of 80% ethanol (vehicle control). Initial assays at 1mg/mL indicated that the IC50s for the top 4 extracts were below 500 µg/mL; so, subsequent assays used 500 µg/mL as the highest concentration. Exosome formulations of these extracts were also tested at the same phytochemical concentrations. After 68–70 h, the cells were co-incubated with 0.5 mg/mL MTT (Avantor, Radnor, PA, USA) for 2–4 h. The media were aspirated, and the MTT formazan crystals were solubilized in DMSO (Avantor, Radnor, PA, USA). The plate was measured at 570 nm on a SpectraMax M2 UV/Vis microplate reader (Molecular Devices, San Jose, CA, USA).

### 2.7. Statistical Analyses

Statistical testing was performed using GraphPad Prism 10.0. Data sets were separately compared with either one-way or two-way ANOVAs with Tukey’s, Dunnett’s, or Šídák’s multiple comparisons tests as indicated. ANOVA and multiple-comparisons test tables are available in Supplemental Tables S1–S23. Some multiple comparison test results are indicated with a compact letter display (CLD) to show statistically similar data (*p* > 0.05), which shares a letter displayed above a given box plot. The results that do not share a CLD letter have a statistically significant difference (*p* < 0.05). Other multiple-comparison test results are indicated with asterisks, which have the following designations: * = *p* < 0.05, ** = *p* < 0.005, *** = *p* < 0.001, **** = *p* < 0.0001; all unlabeled comparisons are ns (*p* > 0.05).

## 3. Results

### 3.1. Phytochemical Content of Apiaceae Spice Ethanolic Extracts

**Figure 2:**
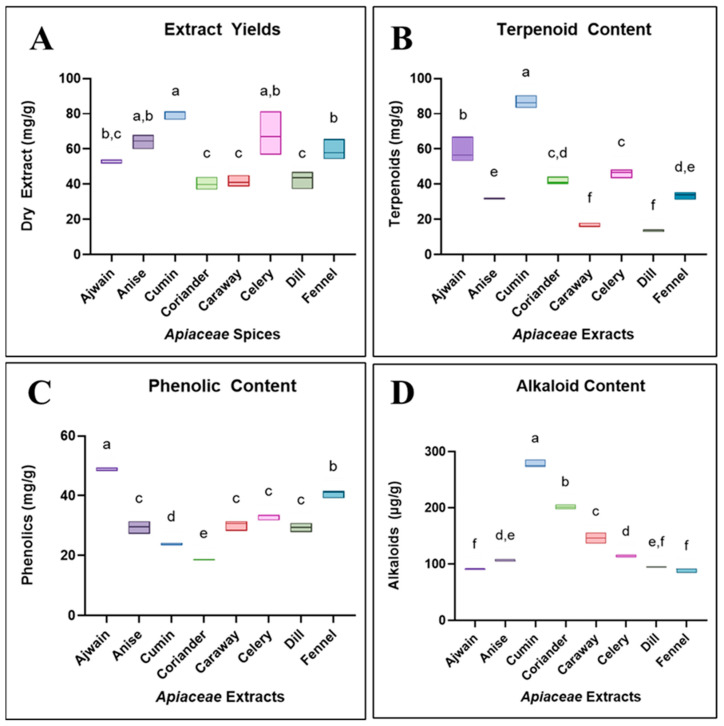
(**A**) *Apiaceae* extract mass recovered (extract (mg)/ground seed (g)); (**B**) quantified total terpenoid content of *Apiaceae* extracts expressed as linalool equivalent (mg)/dry seed extract (g); (**C**) quantified total phenolic content of *Apiaceae* extracts expressed as gallic acid equivalent (mg)/dry seed extract (g); (**D**) quantified total alkaloid content of *Apiaceae* extracts expressed as atropine equivalent (µg)/dry seed extract (g). Values represent the average of three replicates and results are displayed using a boxplot; box height represents data distribution. A compact letter display (CLD) is used to display statistically similar data (*p* > 0.05), which shares a letter displayed above a given box plot. The results that do not share a CLD letter have a statistically significant difference (*p* < 0.05).

*Apiaceae* spice seeds have diverse phytochemistry with significant stores of phenolics, terpenoids, and alkaloids with medicinally relevant properties [9,10,11,34,35]. Phytochemical assays were used to quantify and then compare the contents of each spice extract (Table 1). The *Apiaceae* seeds, on average, yielded between 40 mg/g and 80 mg/g (*w*/*w*; extract/powdered seeds) (Figure 2A). Most of the extracts had similar yields ranging from 60 mg/g to 80 mg/g, but ajwain, caraway, coriander, and dill had lower yields in the 40 mg/g to 50 mg/g range. The total terpenoid content of the extracts was variable among the different *Apiaceae* spices, with the lowest amount of terpenoids found in the dill extract (13.6 ± 2.1 mg/g dry extract) and the highest found in the cumin extract (86.7 ± 3.6 mg/g dry extract) (Figure 2B and Figure S1). The estimated total phenolics found in these extracts were less variable (Figure 2C and Figure S2). Ajwain and fennel had the highest phenolic content (48.7 ± 0.6 mg/g and 40.6 ± 1.3 mg/g dry extract, respectively), while cumin and coriander had the lowest (23.8 ± 0.5 mg/g and 18.5 ± 0.3 mg/g dry extract, respectively). The estimated total alkaloids for most of these extracts were consistently at around 100 µg (atropine equivalent) per g of dry extract (Figure 2D and Figure S3). Cumin contained the highest concentration of alkaloids (277.4 ± 6.5 µg/g dry extract), with three times as much as those of the lowest *Apiaceae* spices, fennel and ajwain (89.1 ± 3.8 µg/g and 90.8 ± 1.0 µg/g dry extract).

These phytochemical assays revealed statistically significant differences between the spices when tested with one-way ANOVAs, all with *p* < 0.001. Statistical groupings calculated with one-way ANOVAs and Tukey’s multiple comparisons of *p* > 0.05 are shown in Figure 2 (Tables S1–S4).

**Figure 3:**
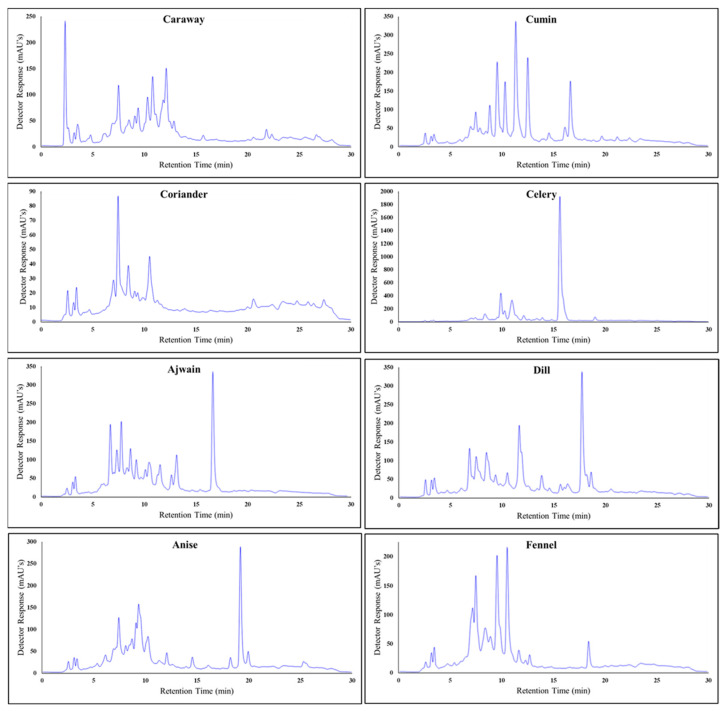
HPLC-DAD chromatograph fingerprints for each *Apiaceae* spice extract (caraway, ajwain, coriander, anise, cumin, celery, fennel, and dill) at 10 mg/mL in 80% ethanol, measured at 280 nm.

Plant extracts represent a large and diverse mixture of phytochemicals that individually interact with each other to produce their combined active effect. The *Apiaceae* family characteristically has a wealth of diverse phenolic content, and many of those phenolics have been found to have anticancer mechanisms [35,36,37,38,39,40]. Since all of the spice extracts had significant stores of phenolics, the similarities and distinctions of the phenolic profiles of the tested spices were examined with HPLC. Unique HPLC-DAD qualitative fingerprints were produced for each extract by monitoring column elutions at 280 nm (Figure 3). These fingerprints represent the phenolic profiles of each extract, and HPLC total peak area measurements from these assays were used as an estimate of phytochemical content for subsequent calculations.

### 3.2. Acid Hydrolysis Impacts Exosome Loading of Apiaceae Spice Extracts

To enhance exosome loading and to assess polarity as a factor that could influence bioactivity, an acid hydrolysis (AH) protocol was optimized to cleave glycosides that can modulate phytochemical bioactivity and polarity. To monitor phytochemical conversion and potential degradation during AH, HPLC was used to measure and compare the total peak area of the processed extracts and AH-extracts. Previous work with exosomal loading of individual compounds reported drug loading results as a ratio of drug mass to exosomal protein. However, plant extracts also contained protein and thus would conflate reporting drug load on the basis of drug load/mg exosomal protein. An alternate method was developed to obtain accurate estimates of drug load based on HPLC analysis.

**Figure 4:**
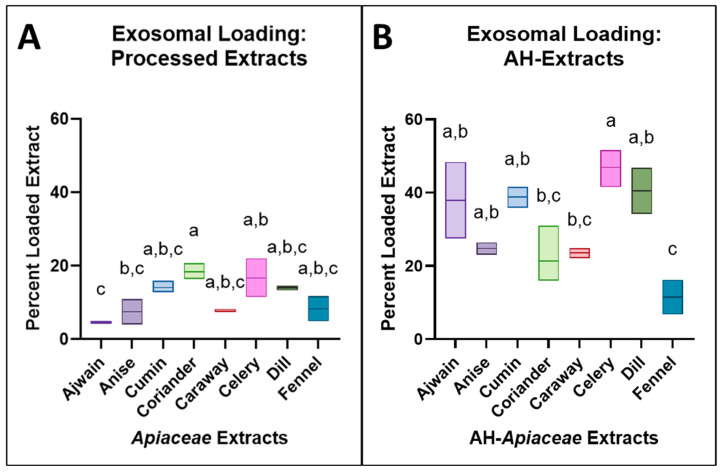
(**A**) Equal volumes of processed extract and processed extract exosomal formulations were assayed and (**B**) equal volumes of AH-extract and AH-extract exosomal formulations (after unbound extract was recovered) were assayed with HPLC-DAD at 280 nm and compared based on total peak area. A compact letter display (CLD) is used to display statistically similar data (*p* > 0.05), which shares a letter displayed above a given box plot. The results that do not share a CLD letter have a statistically significant difference (*p* < 0.05).

Exosomal loading of the processed *Apiaceae* seed extracts, based on HPLC total peak area, indicated phytochemical loading between 4.6% (ajwain) and 18.4% (coriander) (Figure 4A). The loading of the spices was in the range of 5–18%, compared to 10–40% reported for pure molecules of varying hydrophobicity [21]. Coriander and cumin extracts had the highest loading, with 18.4% and 14.1%, respectively, while ajwain and anise had the lowest, with 1.9% and 7.5%, respectively (Figure 4A). Exosome formulations were grouped based on loading potential, with a one-way ANOVA and Tukey’s multiple comparisons with statistically similar peak areas *p* > 0.5 (Figure 4A, Table S5).

**Figure 5:**
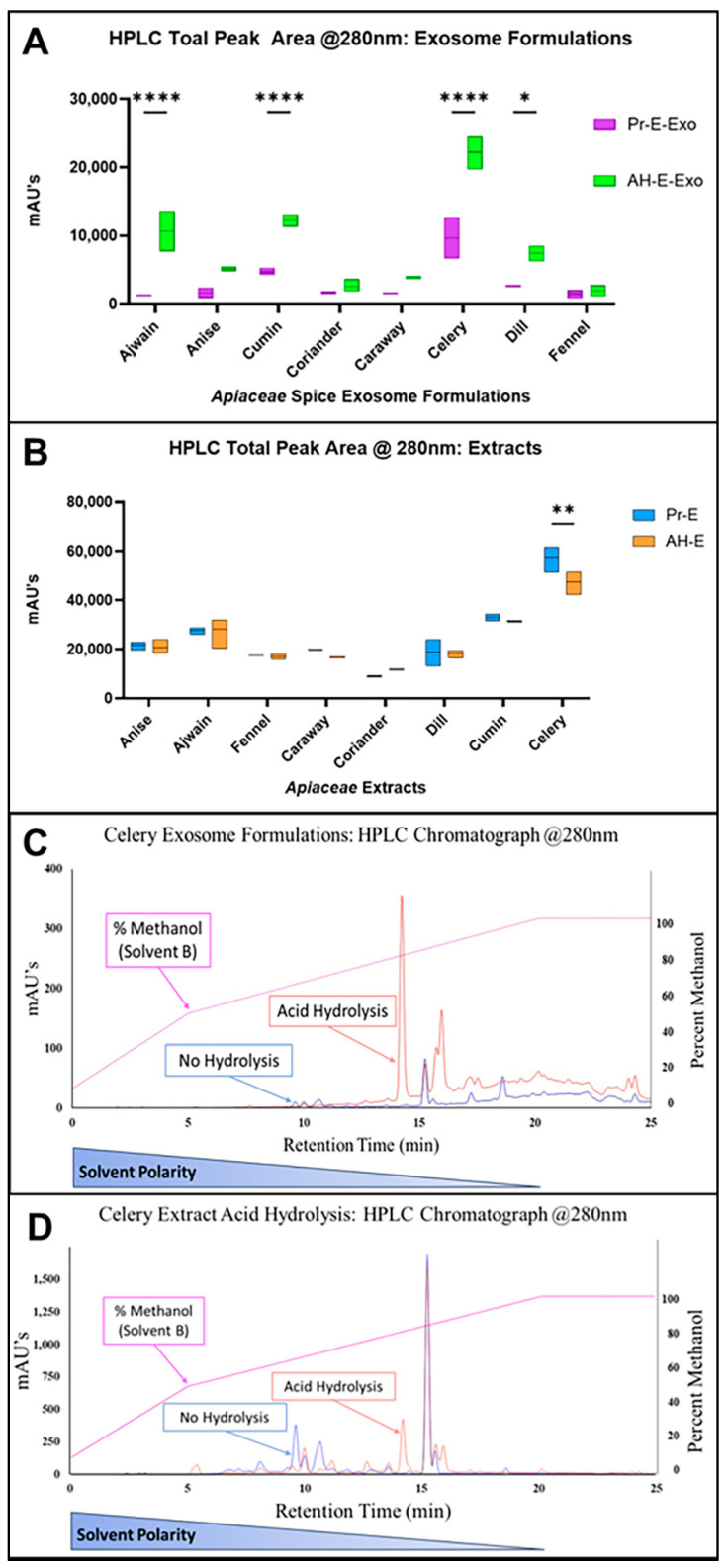
(**A**) Comparison of *Apiaceae* processed extract exosomal formulations (Pr-E-Exo) and AH extract exosomal formulations (AH-E-Exo), where AH significantly increased extract loading for ajwain, cumin, celery, and dill, and (**B**) comparison of processed *Apiaceae* spice extracts (Pr-E) and AH-extracts (AH-E) based on HPLC total peak area. (**C**) HPLC chromatographs showing recovered phytochemicals from processed celery (PR-E-Exo: blue) overlaid with AH-celery (AH-E-Exo: orange) exosomal formulations and (**D**) processed celery (PR-E: blue) overlaid with AH-celery (AH-E: orange) extracts. Methanol solvent gradient (%) is displayed in pink to highlight the relatively lower polarity of compound peaks as they elute. The arrows are pointing to the same peaks in both chromatographs in (**C**,**D**), highlighting that each peak is not loaded equally. Multiple-comparison test results are indicated with asterisks, which have the following designations: * = *p* < 0.05, ** = *p* < 0.005, **** = *p* < 0.0001; all unlabeled comparisons are ns (*p* > 0.05).

Exosomal loading of the AH-extracts indicated increased phytochemical loading ranging from 11.5% for the lowest (AH-fennel) and 46.7% for the highest (AH-celery) (Figure 4B, Table S6). AH-extract loading of ajwain, cumin, celery, and dill was significantly improved relative to exosomal loading of processed extracts, while anise, coriander, caraway, and fennel showed no significant difference. HPLC total peak areas for recovered exosome formulations of all AH extracts were evaluated using a two-way ANOVA and found to be statistically significant in comparison to the processed extracts in half of the samples (Figure 5A, Table S7). AH resulted in minimal total peak area degradation with no significant differences (*p* > 0.05), except for celery (*p* = 0.002), which experienced some loss of total peak area (Figure 5B and Table S8).

HPLC chromatograph fingerprints of the AH-*Apiaceae* seed extracts show a shift in the phytochemical profiles due to glycoside conversions to aglycones, for celery (Figure 5D) and all other spices (Figure S4). A qualitative example of improved exosomal loading of processed celery extract and celery AH-extract shows a dramatic impact (Figure 5C) and the impact was similar for all spices in our studies (see Figure S5).

**Figure 6:**
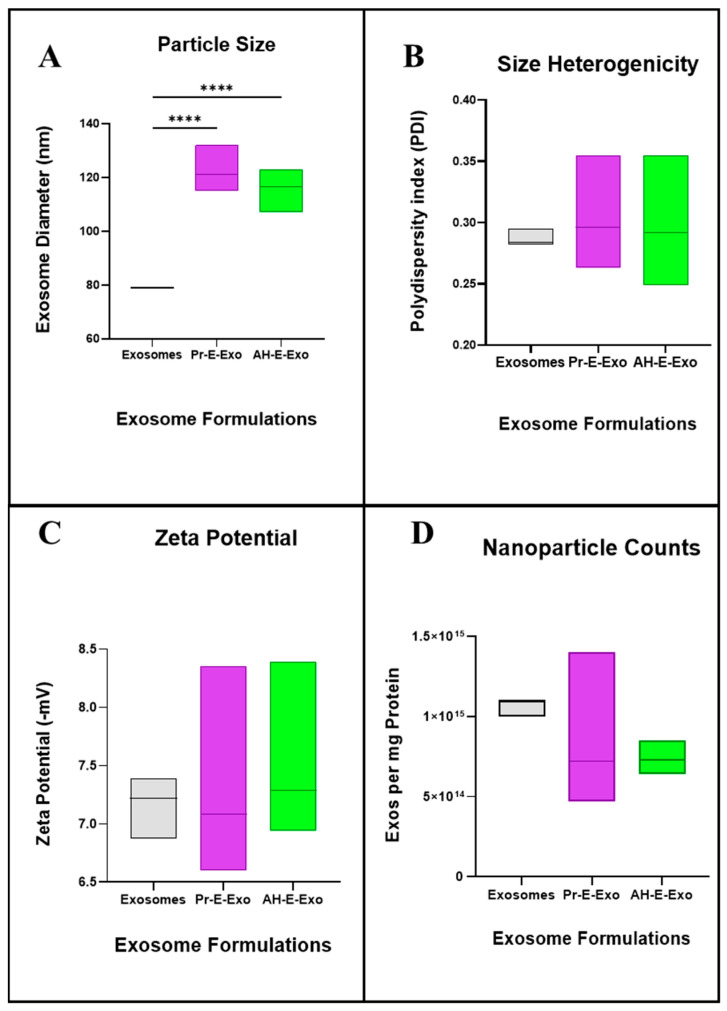
Characterization of exosomal formulations (**A**) exosome size, (**B**) polydispersity index (PDI), (**C**) zeta potential, and (**D**) particle counts. Multiple-comparison test results are indicated with asterisks, which have the following designations: **** = *p* < 0.0001; all unlabeled comparisons are ns (*p* > 0.05).

To determine the potential impacts of loaded *Apiaceae* spice extracts on the characteristics of bovine milk exosomes, size, charge, and PDI were assessed (Figure 6). The processed and AH-*Apiaceae* exosome formulations were not significantly different from each other (122.0 ± 5.3 nm and 116.5 ± 5.4 nm, respectively) but were larger on average than control (unloaded) bovine milk exosomes (79.0 nm) (Figure 6A). The polydispersity index (PDI) is a measure of particle size heterogeneity that ranges from 0 to 1, where <0.1 is highly monodisperse, 0.1–0.7 is moderately polydisperse, >0.7 is highly polydisperse (or heterogeneous), and a PDI of less than 0.3 is considered sufficiently monodisperse for lipid-based nanoparticle drug delivery formulations [41]. *Apiaceae* AH and processed extract loaded exosomes had an average PDI of 0.293 ± 0.037 and 0.301 ± 0.030, respectively, compared to unloaded exosomes (0.287 ± 0.007 PDI) (Figure 6B).

The zeta potential is a measure used to assess colloidal stability, which is influenced in part by the repulsive force of the negatively charged nanoparticles. Bovine milk exosomes tend to have low zeta potential/colloidal stability. Bovine milk exosomes had a zeta potential (surface charge) of −7.16 mV ± 0.27 mV, while the AH and processed *Apiaceae* extract loaded exosomes had similar zeta potentials with an average of −7.45 ± 0.56 mV and −7.25 ± 0.56 mV, respectively (Figure 6C). Individual exosome counts were obtained with a Particle Metrix ZetaView. Exosomes were tracked and counted on an equal exosome protein basis to assess the potential impact of loading on exosome quantities. There was no significant difference (*p* > 0.05) in the number of exosomes per mg protein for the processed extract and AH-extract loaded exosomes with 8.38 × 10^14^ ± 3.29 × 10^14^ and 7.41 × 10^14^ ± 7.51 × 10^13^ nanoparticles per mg, respectively. The unloaded exosomes had an average of 1.07 × 10^15^ ± 5.77 × 10^13^ nanoparticles per mg exosomal protein (Figure 6D). None of the individual *Apiaceae* exosome formulations differed significantly from their pooled means shown above (Figure S6 and Tables S9–S12).

### 3.3. The Antiproliferative Effects of Apiaceae Spice Extracts Are Impacted by Acid Hydrolysis

**Figure 7:**
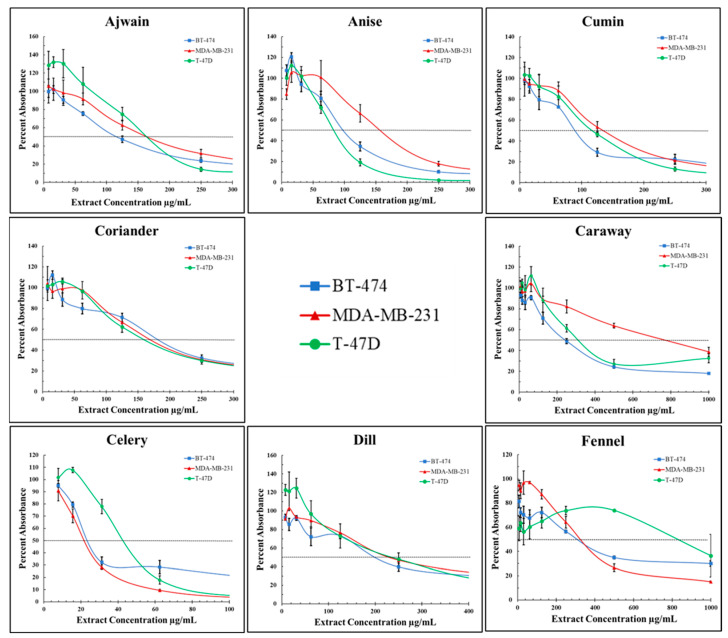
Cellular proliferation dose response curves of BT-474, MDA-MB-231, and T-47D cells treated with initial *Apiaceae* spice extracts in concentrations ranging from 8 to 1000 µg/mL dry extract. Values are inhibition percentage relative to corresponding ethanol controls, with each point representing the average of two or three replicates, and error bars representing standard deviation. The *X*-axis is zoomed in to highlight differences in IC50s.

**Figure 8:**
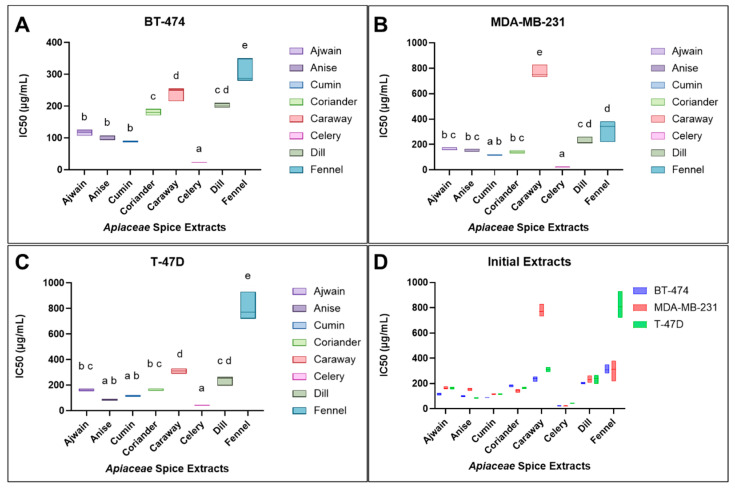
Calculated IC50s for (**A**) BT-474, (**B**) MDA-MB-231, and (**C**) T-47D breast cancer cells treated with *Apiaceae* spice extracts. Each box plot represents three replicates. (**D**) *Apiaceae* spice extract IC50s compared across the three cancer cell types. A compact letter display (CLD) is used to display statistically similar data (*p* > 0.05), which shares a letter displayed above a given box plot. The results that do not share a CLD letter have a statistically significant difference (*p* < 0.05).

To compare the antiproliferative effects of the *Apiaceae* extracts across several breast cancer cell lines, dose response curves were produced (Figure 7) and used to calculate IC50s (inhibitory concentration-50%) (Table 2) for each initial *Apiaceae* seed extract (In-E) in BT-474 (Figure 8A), MDA-MB-231 (Figure 8B), and T-47D (Figure 8C) breast cancer cells. All initial *Apiaceae* spice extracts were found to inhibit the proliferation of breast cancer cells with differential efficacy, providing a rank order from most to least effective, based on IC50 calculations averaged across all three cell types: celery > cumin > anise > ajwain > coriander > dill > caraway > fennel extracts (Table 2 and Figure 8). Statistical analysis of the IC50s of the extracts was calculated using a one-way ANOVA for each cell line, with groupings based on Tukey’s multiple comparisons test with statistically similar extracts *p* > 0.05, where (a) represents the lowest IC50s and most effective extract, while (b) has a relatively higher IC50 and is less effective at inhibiting cellular proliferation, and (e) has the highest IC50s with the least activity (Figure 8A–C and Tables S13–S15). Figure 8D compiles these data into a single graph for comparisons of the effect of *Apiaceae* spice extracts across the breast cancer cell types tested.

**Figure 9:**
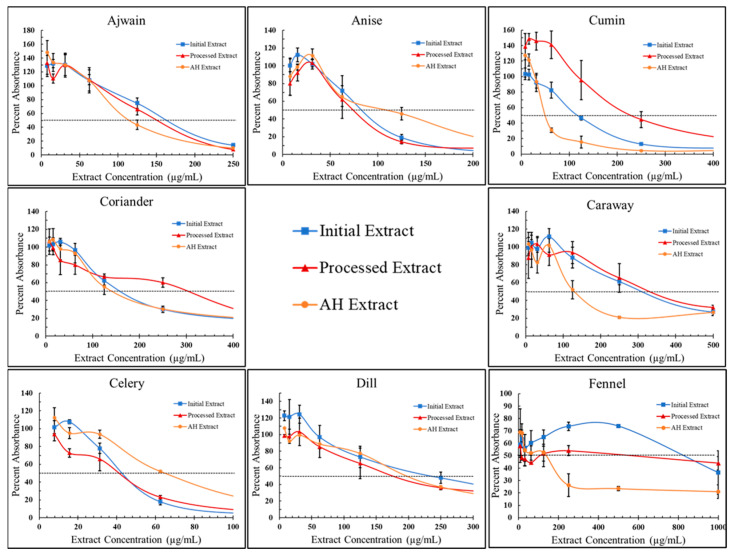
Cellular proliferation dose response curves of T-47D cells treated with *Apiaceae* spice extracts in concentrations ranging from 8 to 1000 µg/mL dry extract. Processed extracts were exposed to the process of AH without acid. Values are inhibition percentage relative to corresponding ethanol controls, each point represents the average of two or three replicates, and error bars represent standard deviation. *X*-axis zoomed into to highlight IC50s.

**Figure 10:**
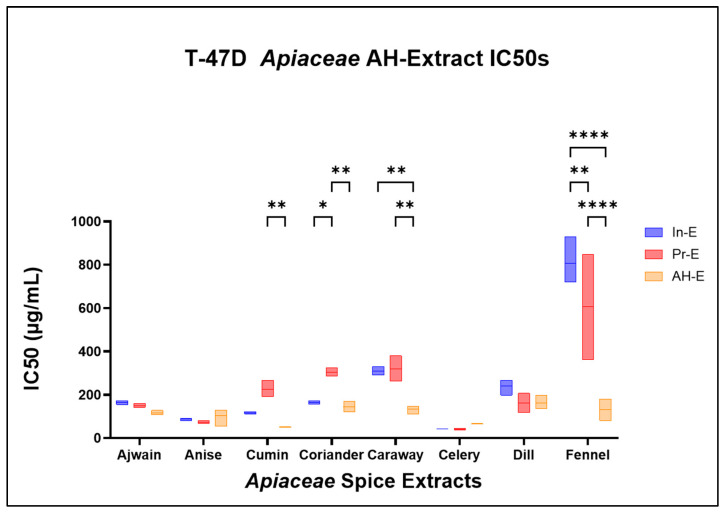
Calculated IC50s for T-47D breast cancer cells treated with *Apiaceae* spice extracts. Each box plot represents two or three replicates. Multiple-comparison test results are indicated with asterisks, which have the following designations: * = *p* < 0.05, ** = *p* < 0.005, **** = *p* < 0.0001; all unlabeled comparisons are ns (*p* > 0.05).

After establishing the broad antiproliferative effects of *Apiaceae* spice extracts on breast cancer cell lines, acid hydrolysis was used to cleave glycosidic compounds to potentially improve these observed effects. To determine the effects of AH on *Apiaceae* extract antiproliferative activity, dose response curves were produced with T-47D cells only (Figure 9) and used to calculate IC50s (inhibitory concentration of 50%) (Table 3) for each initial *Apiaceae* seed extract (In-E) and compared with AH-extracts (AH-E) and “processed” extracts that were exposed to the same process as AH but without acid (Pr-E). The impact of acid hydrolysis, with or without acid, on antiproliferative bioactivity was mixed (Table 3 and Figure 10). The process of acid hydrolysis, with or without acid, did not significantly impact the IC50s of ajwain (IE: 163.7 ± 9.5 µg/mL, Pr-E: 149.7 ± 9.6 µg/mL, AH-E 115.7 ± 11.0 µg/mL), anise (IE: 87.0 ± 6.1 µg/mL, Pr-E: 73.7 ± 6.5 µg/mL, AH-E 102 ± 41.6 µg/mL), and celery (IE42.7 ± 0.6 µg/mL, Pr-E: 40.3 ± 4.9 µg/mL, AH-E 65.7 ± 1.1 µg/mL) extracts compared to their initial extract counterparts (Figure 10). The IC50 of the processed cumin extract was increased compared to the initial extract (IE: 116.0 ± 6.0 µg/mL, Pr-E: 225.7 ± 38.8 µg/mL), while the addition of acid in the AH-extract lowered the IC50 by roughly half compared to the initial extract (AH-E: 49.7 ± 2.1 µg/mL). Coriander IC50s were increased in the processed extracts (IE: 162.7 ± 9.1 µg/mL, Pr-E: 303.7 ± 19.6 µg/mL) and brought back to similar IC50s as the initial extract with the addition of acid (AH-E: 144 ± 25.1 µg/mL). The processed caraway extract was not significantly different compared to the initial extract (IE: 310.0 ± 20.0 µg/mL, Pr-E: 318.3 ± 59.2 µg/mL), but the AH-extract had a significantly lower IC50 (AH-E: 133.3 ± 19.7 µg/mL). Dill bioactivity was increased in both the processed extract and the AH-extract (IE: 240.0 ± 37.6 µg/mL, Pr-E: 161 ± 45.0 µg/mL, AH-E 162.3 ± 32.3 µg/mL), and the addition of acid did not significantly impact the IC50. Fennel extract activity was increased from the initial extracts (806.7 ± 109.7 µg/mL) to the processed extracts (605.0 ± 343.7 µg/mL) and further increased in the AH-extracts (130.0 ± 50.0 µg/mL). Statistical analyses were calculated using a two-way ANOVA with Tukey’s multiple comparisons revealed significant differences influenced by both the *Apiaceae* spice and the extract processes (Table S16).

### 3.4. Exosomal Delivery Enhances Antiproliferative Effects of Apiaceae Spice Extracts

**Figure 11:**
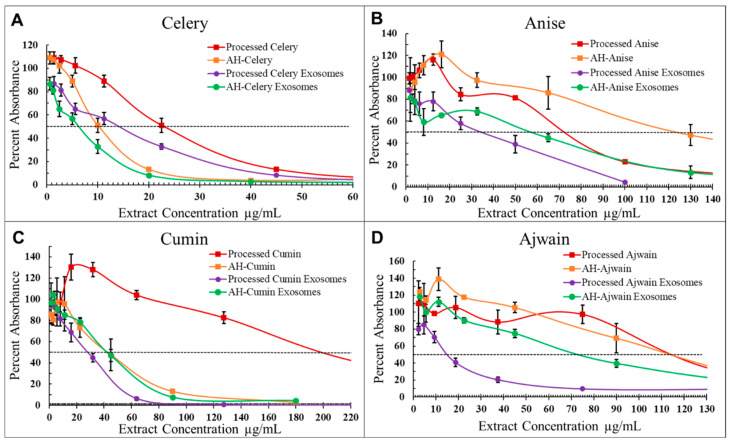
Averaged processed extracts (Pr-E: red), AH-extracts (AH-E: orange), processed extract exosome formulation (Pr-E-Exo: purple), and AH-extract exosome formulations (AH-E-Exo: green) MTT dose response curves for T-47D breast cancer cells treated with (**A**) celery, (**B**) anise, (**C**) cumin, and (**D**) ajwain. Values are inhibition percentage relative to corresponding ethanol controls, each point represents the average of three replicates, and error bars represent standard deviation. *X*-axis zoomed into to highlight IC50s.

**Figure 12:**
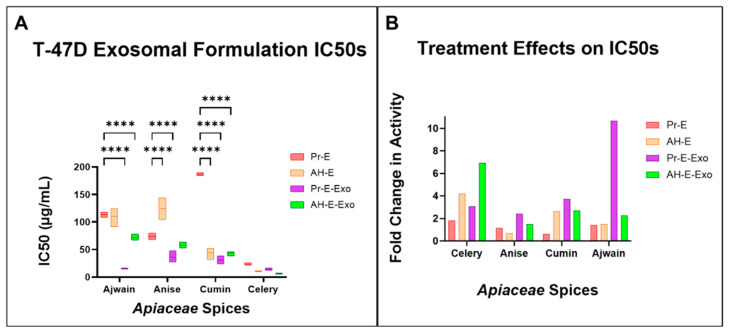
(**A**) IC50 values for the top four *Apiaceae* spice extracts, directly comparing processed extract (Pr-E), AH-extract (AH-E), processed extract exosome formulations (Pr-E-Exo), and AH-extract exosome formulations (AH-E-Exo). (**B**) Fold change in activity compared to their initial extract counterparts for the top four *Apiaceae* spices, comparing the effects of acid hydrolysis and exosomal delivery; these are the data from Table 4 and Figure 12A compared to the IC-50s of the initial extracts from Table 2 and Figure 8C, displayed in this manner to highlight the effects of AH compared to exosomal delivery. Multiple-comparison test results are indicated with asterisks, which have the following designations: **** = *p* < 0.0001; all unlabeled comparisons are ns (*p* > 0.05).

To assess the effect of exosomal delivery, the *Apiaceae* spice extracts with the four lowest IC50 values for AH-seed extracts were further assayed for antiproliferative effects in exosomal formulations. Dose response curves were produced (Figure 11) and used to calculate IC50s (inhibitory concentration of 50%) (Table 4 and Figure 12A) for each processed *Apiaceae* seed extract (Pr-E) and compared with AH-extracts (AH-E), processed extract exosomal formulations (Pr-E-Exo) and AH-extract exosomal formulations (AH-E-Exo). The exosomal formulations showed dose-dependent activity, with the greatest reduction in cell proliferation with AH-celery, processed celery, and AH-ajwain (Table 4, Figure 11 and Figure 12A). Exosomal delivery significantly enhanced the effectiveness for most of the exosomal spice formulations compared to their unloaded extract counterparts except for AH-anise, AH-celery, and processed celery (Figure 11 and Figure 12A). A full two-way ANOVA table with Dunnet’s multiple comparisons test is shown in Table S17.

Exosomal formulations increased the effectiveness of all spice extracts when compared to their extract counterparts except for AH-cumin, which had a similar IC50 values with either delivery method (Table 4 and Figure 12A). Exosomal delivery improved the IC50s the most for the processed cumin and ajwain formulations (6.0× and 7.4× lower, respectively), while AH-cumin and AH-ajwain formulations had the lowest improvements (1.0× and 1.5×, respectively) (Table 4 and Figure 12A). Celery and anise formulations had similar improvements when compared to either their processed or AH-extract counterparts (celery Pr-E-Exo: 1.7×, AH-E-Exo: 1.7×; anise Pr-E-Exo: 2.1×, AH-E-Exo: 2.2×).

To highlight the effects of AH and exosomal delivery on *Apiaceae* spice extract antiproliferative activity, these data from Table 4 and Figure 12A are displayed as a fold change in activity over the initial extracts (In-E) from Table 3 and Figure 10, shown in Figure 12B. Compared to the IC50s of the initial extracts, AH-celery (4.2×), AH-celery exosomes (6.9×), and processed ajwain exosomes (10.7×) had the greatest overall improvements (Figure 12B).

## 4. Discussion

The *Apiaceae* spice family includes numerous plant species with rich phytochemical content and anticancer bioactivities [10,11,34,35,42]. Ethanolic extracts of all *Apiaceae* spices assayed demonstrated measurable terpenoid, alkaloid, and phenolic contents. These extracts exhibited antiproliferative effects against BT-474, MDA-MB-231, and T-47D cell cultures, with a rank order of bioactivity from highest to lowest of celery > cumin > anise > ajwain > coriander > dill > caraway > fennel.

To further improve efficacy, two distinct variables, acid hydrolysis (AH) and exosome nanoparticle delivery, were investigated. *Apiaceae* spice extracts were acid-hydrolyzed to remove glycosides and reduce phytochemical polarity which has a significant effect on exosomal loading. Each AH-extract was tested for altered antiproliferative bioactivity in comparison to the initial extracts and the processed extracts (AH extract controls, without the acid) with T-47D cells. Acid hydrolysis of cumin, caraway, fennel, and dill extracts displayed lower IC50 values compared to the initial extracts, whereas AH-extracts of ajwain, anise, celery, and coriander showed no significant change in bioactivity compared to the initial extracts. For cumin and coriander, the processed extracts had higher IC50s than the initial extracts, but their AH extracts either restored similar IC50s (coriander) or significantly lowered the IC50s (cumin).

The passive loading potential of compounds through room-temperature incubation with exosomes is largely impacted by the hydrophobicity of the molecules being loaded, low- to medium-molecular-weight hydrophobic phytochemicals like curcumin, cucurbitacin, celastrol, and paclitaxel have been successfully loaded, while hydrophilic molecules often require some disruption of the exosomal membrane to improve loading [13,21]. Since plant-specialized metabolites tend to be glycosylated, initial ethanolic spice extracts were expected to have relatively high polarity compared to their aglycone forms [43,44]. The observed low loading potential in initial and processed extracts is likely due to the high polarity caused by the remaining glycosyl groups. In addition to improving the antiproliferative activity of some extracts, acid hydrolysis increased the phytochemical loading potential of all *Apiaceae* seed extracts into bovine milk exosomes. The lowest improvement in loading was for the AH-coriander extract, with exosome loading increasing by 1.2-fold (from 18.4% to 21.4%) and the most substantial exosome loading improvement was for the AH-ajwain extract loading by 8.2-fold (from 4.6% to 37.9%). AH-celery had the highest overall loading, with 46.9% AH-extract loaded into exosomes.

The four extracts (anise, ajwain, cumin, and celery) with the highest bioactivity were assayed for their ability to inhibit the cellular proliferation of T-47D breast cancer cell cultures with exosomal delivery in comparison to free AH-extracts and processed extracts. Exosomal delivery of *Apiaceae* phytochemicals increased the effectiveness of all exosomal formulations assayed compared to the initial extracts. For all extracts assayed, except AH-cumin, the exosome formulations consistently improved bioactivity compared to the initial extracts regardless of acid hydrolysis. Phytochemical extract loading in bovine milk exosomes is enhanced with acid hydrolysis and AH-extract formulations require fewer exosome nanoparticles to achieve the same free extract dose. The inherent antiproliferative bioactivities of *Apiaceae* spices can thus be increased with acid hydrolysis and further enhanced with exosomal delivery, but this is species-specific and likely requires optimization for each targeted species.

The results of these experiments indicate that complex plant extracts can be loaded into bovine milk exosomes and that this process can be improved with acid-hydrolyzed extracts. We have demonstrated that acid hydrolysis can impact plant extract bioactivity and improve the extract’s exosomal loading potential. These findings represent a step toward improving the loading of plant compounds that demonstrate interesting activities but suffer from poor oral bioavailability. These processes could be used to lower their required doses and improve their systemic distribution. Many chemotherapies are associated with side effects, and natural plant treatments, which tend to have less severe side effects, delivered with bovine milk exosomes could be an alternative treatment for patients who cannot tolerate traditional treatments.

This research represents an exploratory study to determine which *Apiaceae* spices warrant further investigation when acid hydrolysis and exosomal delivery are applied to ethanolic seed extracts with a focus on antiproliferative effects in breast cancer cell lines. While this research does indicate that some *Apiaceae* spices have antiproliferative effects in breast cancer cell models, this study is limited by not examining these effects in vivo, where the impacts on systemic bioavailability could be assessed and antiproliferative effects confirmed in an animal model. Additionally, HPLC-DAD was employed as a qualitative technique in this study to monitor the effects of acid hydrolysis and exosomal loading on the phenolic fingerprints of each spice extract and exosomal formulation, but further assays are needed to identify the mixture of phytochemicals that contribute to their antiproliferative effects.

Future research will focus on phytochemical identifications to determine the active compounds in the most effective spice formulations, how they are affected by acid hydrolysis, and how this impacts individual phytochemical loading in bovine milk exosomes. After the phytochemical assessments, future research will focus on in vivo animal studies, comparisons with non-cancerous cell models, and mechanistic assays in cell cultures to confirm these preliminary results.

## Figures and Tables

**Table 1:** *Apiaceae* spice extraction and phytochemical content.

Spice	Extract Yield ^1^	Alkaloids ^2^	Phenolics ^3^	Terpenoids ^4^
Ajwain	52.6 ± 1.1	90.8 ± 1.0	48.7 ± 0.6	58.9 ± 7.1
Anise	64.1 ± 4.1	106.2 ± 2.0	29.5 ± 2.1	31.9 ± 0.5
Cumin	79.6 ± 2.6	277.4 ± 6.5	23.8 ± 0.5	86.7 ± 3.6
Coriander	40.3 ± 3.6	201.3 ± 3.9	18.5 ± 0.3	41.8 ± 2.2
Caraway	41.6 ± 3.2	146.2 ± 9.3	30.1 ± 1.7	17.1 ± 1.3
Celery	68.3 ± 12.3	114.2 ± 2.0	32.88 ± 1.0	46.1 ± 2.4
Dill	42.6 ± 4.9	94.6 ± 1.0	29.38 ± 1.5	13.6 ± 0.7
Fennel	59.2 ± 5.8	89.1 ± 3.8	40.6 ± 1.3	33.6 ± 2.1

^1^ mg/g *w*/*w* dry extract/ground seeds; ^2^ µg/g *w*/*w* atropine equivalent/dry extract; ^3^ mg/g *w*/*w* gallic acid equivalent/dry extract; ^4^ mg/g *w*/*w* linalool equivalent/dry extract.

**Table 2:** Initial *Apiaceae* spice extract IC50s (µg/mL) for BT-474, MDA-MB-231 and T-47D breast cancer cells.

Spice	BT-474	MDA-MB-231	T-47D
Ajwain	117.0 ± 9.5	163.3 ± 11.9	163.7 ± 9.5
Anise	98.0 ± 7.8	156.0 ± 12.3	87.0 ± 6.1
Cumin	89.0 ± 3.0	117.3 ± 5.7	116.0 ± 6.0
Coriander	180.8 ± 9.8	114.7 ± 13.1	162.7 ± 9.1
Caraway	240.0 ± 21.8	770.0 ± 5	310.0 ± 20.0
Celery	23.4 ± 1.0	21.8 ± 1.0	42.7 ± 0.6
Dill	201.7 ± 7.6	228.7 ± 29.6	240.0 ± 37.6
Fennel	305.7 ± 38.6	313.7 ± 84.2	806.7 ± 109.7

**Table 3:** *Apiaceae* spice extract IC50s (µg/mL) for T-47D breast cancer cells.

Spice	Initial Extract	Processed Extract ^1^	AH-Extract
Ajwain	163.7 ± 9.5	149.7 ± 9.6	115.7 ± 11.0
Anise	87.0 ± 6.1	73.7 ± 6.5	102 ± 41.6
Cumin	116.0 ± 6.0	225.7 ± 38.8	49.7 ± 2.1
Coriander	162.7 ± 9.1	303.7 ± 19.6	144 ± 25.1
Caraway	310.0 ± 20.0	318.3 ± 59.2	133.3 ± 19.7
Celery	42.7 ± 0.6	40.3 ± 4.9	65.7 ± 1.1
Dill	240.0 ± 37.6	161 ± 45.0	162.3 ± 32.3
Fennel	806.7 ± 109.7	605.0 ± 343.7	130.0 ± 50.0

^1^ Processed extracts were exposed to the process of AH without acid.

**Table 4:** *Apiaceae* extracts and exosomal formulations IC50 (µg/mL) for T-47D breast cancer cells.

Spice	Processed Extract ^1^	AH-Extract	Processed Extract ^1^ Exo	AH Extract Exo
Ajwain	113.7 ± 5.1	109.7 ± 17.2	15.3 ± 1.2	71.3 ± 5.9
Anise	73.5 ± 5.8	124.7 ± 20.0	35.8 ± 10.5	57.3 ± 5.6
Cumin	186.7 ± 3.1	44.2 ± 11.1	31.0 ± 7.0	43.0 ± 4.36
Celery	23.2 ± 2.5	10.2 ± 1.2	13.8 ± 2.0	6.2 ± 0.8

^1^ Processed extracts were exposed to the process of AH without acid.

## Data Availability

The original contributions presented in the study are included in the article/Supplementary Materials, further inquiries can be directed to the corresponding author.

## Supplementary Materials

The following supporting information can be downloaded at: https://www.mdpi.com/article/10.3390/foods13172811/s1. Figure S1: Linalool standard curve, Figure S2: Gallic acid standard curve, Figure S3: Atropine standard curve, Figure S4: Overlaid HPLC-DAD chromatographs for each *Apiaceae* spice at 10 mg/mL dry extract, Figure S5: Overlaid HPLC-DAD chromatographs for each spice exosomal formulation, 5x concentrated to highlight the smaller peaks, Figure S6: Individual AH-*Apiaceae* exosome formulation characterizations, Table S1: One-way ANOVA table for extract yields, Table S2: One-way ANOVA table for terpenoid assay, Table S3: One-way ANOVA table for phenolics assay, Table S4: One-way ANOVA table for alkaloid assay, Table S5: One-way ANOVA table for loading% calculations, Table S6: One-way ANOVA table for AH-extract loading% calculations, Table S7: Two-way ANOVA table for AH exosome formulations’ HPLC measurements, Table S8: Two-way ANOVA table for acid hydrolysis extract HPLC measurements, Table S9: One-way ANOVA table for exosome size measurements, Table S10: One-way ANOVA table for exosome PDI measurements, Table S11: One-way ANOVA table for exosome zeta potential measurements, Table S12: One-way ANOVA table for exosome particle count measurements, Table S13: One-way ANOVA table for *Apiaceae* initial extract IC50s in BT-474, Table S14: One-way ANOVA table for *Apiaceae* initial extract IC50s in MDA-MB-231, Table S15: One-way ANOVA table for *Apiaceae* initial extract IC50s in T-47D, Table S16. Two-way ANOVA table for AH-Extract IC50 Calculations in T-47D , Table S17: Two-way ANOVA Table for AH extract exosome formulation IC50 calculations, Table S18: *Apiaceae* spice extract HPLC total peak area data, Table S19: *Apiaceae* spice exosome formulation HPLC total peak area data, Table S20: MTT Data Proliferation Inhibitory Concentration 50% (IC50) µg/mL: Initial Apiaceae Spice Extracts, Table S21: MTT Data T47-D Proliferation Inhibitory Concentration 50% (IC50) µg/mL: AH-Apiaceae Spice Extracts, Table S22: MTT Data T47-D Proliferation Inhibitory Concentration 50% (IC50) µg/mL: AH-Extract Exosome Formulations, Table S23: AH-extract exosome formulation size data, Table S24: AH-extract exosome formulation PDI data, Table S25: AH-extract exosome formulation zeta potential data, Table S26: AH-Extract exosome formulation nanoparticles per mg protein data.
