# Supplementary material for: Exosomal Delivery Enhances the Antiproliferative Effects of Acid-Hydrolyzed Apiaceae Spice Extracts in Breast Cancer Cells
Source: Foods. 2024 Sep 4;13(17):2811. doi: 10.3390/foods13172811 (PMC11395330; doi:10.3390/foods13172811)
Supplement: Supplementary file 1 [file foods-13-02811-s001.zip › foods-3138646-supplementary.pptx]

## Slide 1
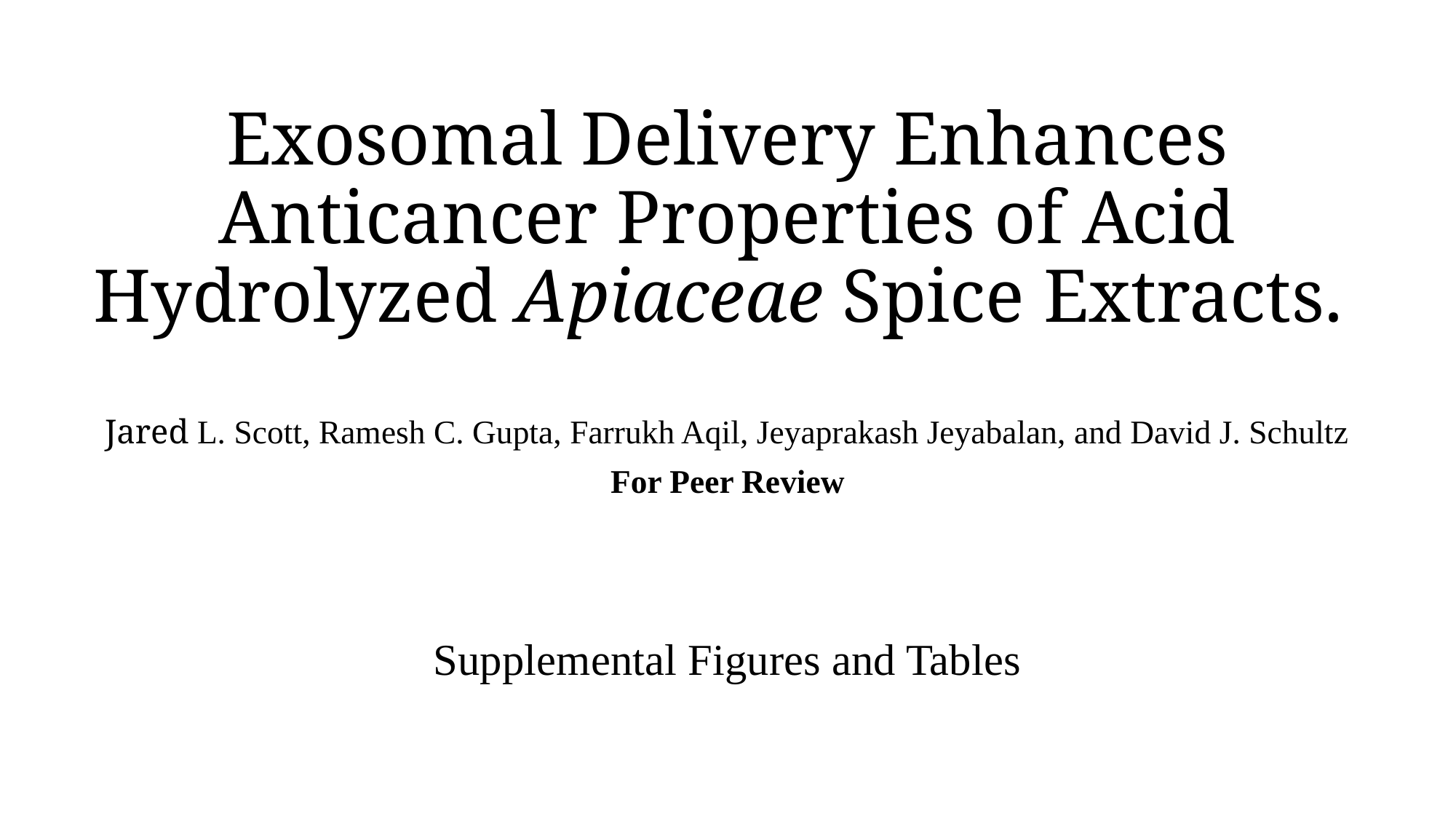

# Exosomal Delivery Enhances Anticancer Properties of Acid Hydrolyzed Apiaceae Spice Extracts.
Jared L. Scott, Ramesh C. Gupta, Farrukh Aqil, Jeyaprakash Jeyabalan, and David J. Schultz
For Peer Review
Supplemental Figures and Tables

## Slide 2
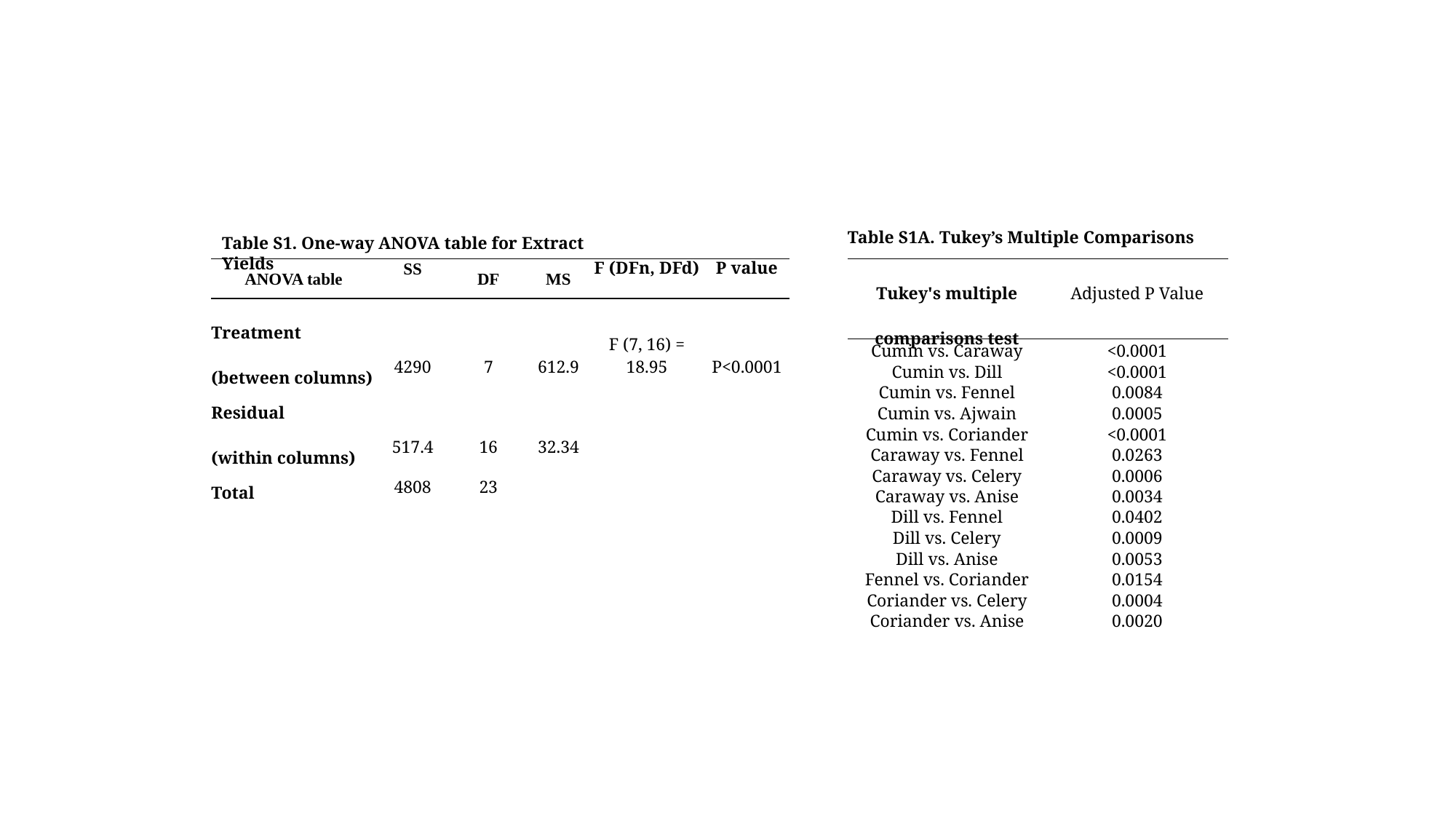

Table S1A. Tukey’s Multiple Comparisons
Table S1. One-way ANOVA table for Extract Yields
| ANOVA table | SS | DF | MS | F (DFn, DFd) | P value |
| --- | --- | --- | --- | --- | --- |
| Treatment (between columns) | 4290 | 7 | 612.9 | F (7, 16) = 18.95 | P<0.0001 |
| Residual (within columns) | 517.4 | 16 | 32.34 | | |
| Total | 4808 | 23 | | | |
| Tukey's multiple comparisons test | Adjusted P Value |
| --- | --- |
| Cumin vs. Caraway | <0.0001 |
| Cumin vs. Dill | <0.0001 |
| Cumin vs. Fennel | 0.0084 |
| Cumin vs. Ajwain | 0.0005 |
| Cumin vs. Coriander | <0.0001 |
| Caraway vs. Fennel | 0.0263 |
| Caraway vs. Celery | 0.0006 |
| Caraway vs. Anise | 0.0034 |
| Dill vs. Fennel | 0.0402 |
| Dill vs. Celery | 0.0009 |
| Dill vs. Anise | 0.0053 |
| Fennel vs. Coriander | 0.0154 |
| Coriander vs. Celery | 0.0004 |
| Coriander vs. Anise | 0.0020 |

## Slide 3
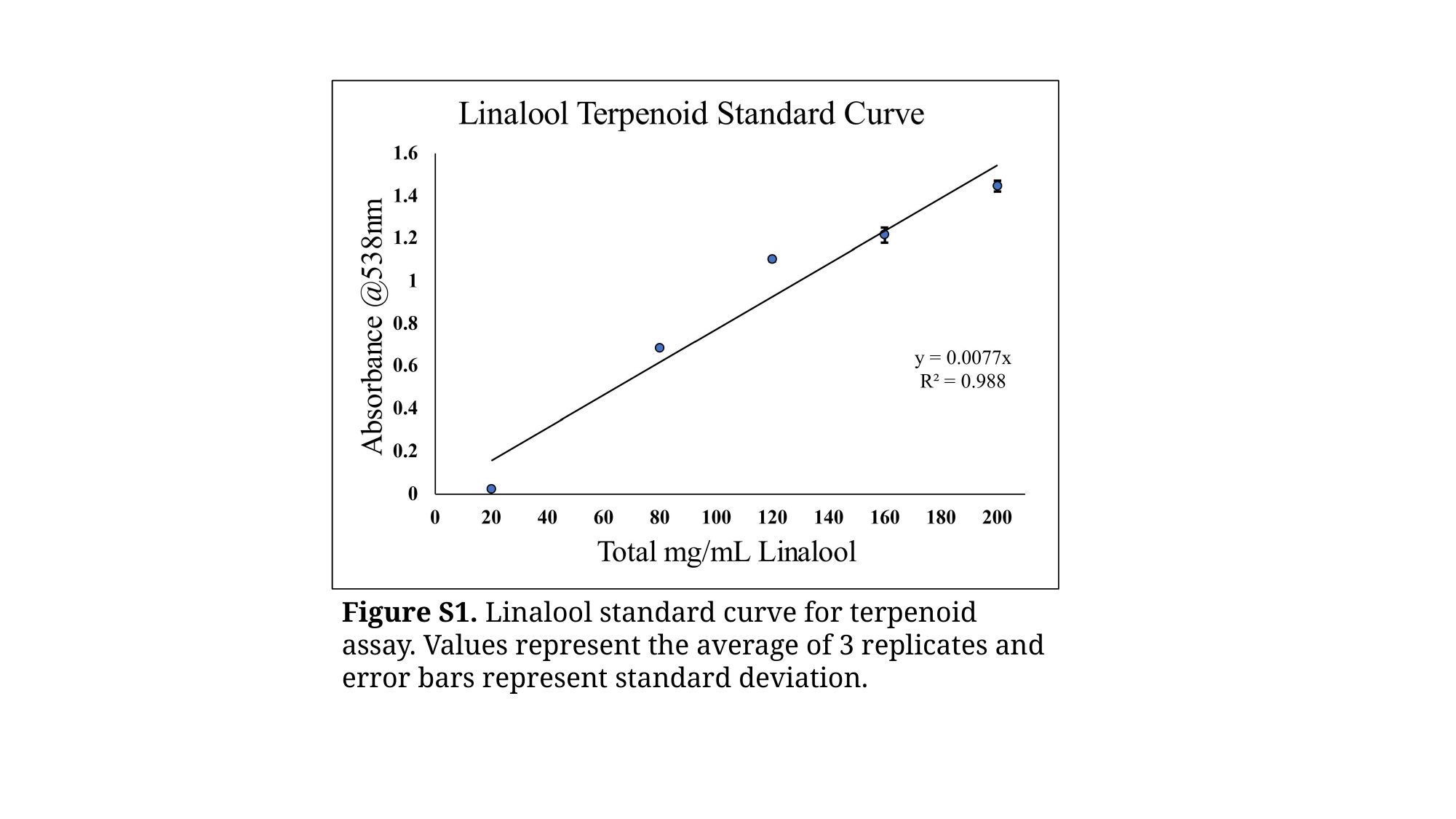

Figure S1. Linalool standard curve for terpenoid assay. Values represent the average of 3 replicates and error bars represent standard deviation.

## Slide 4
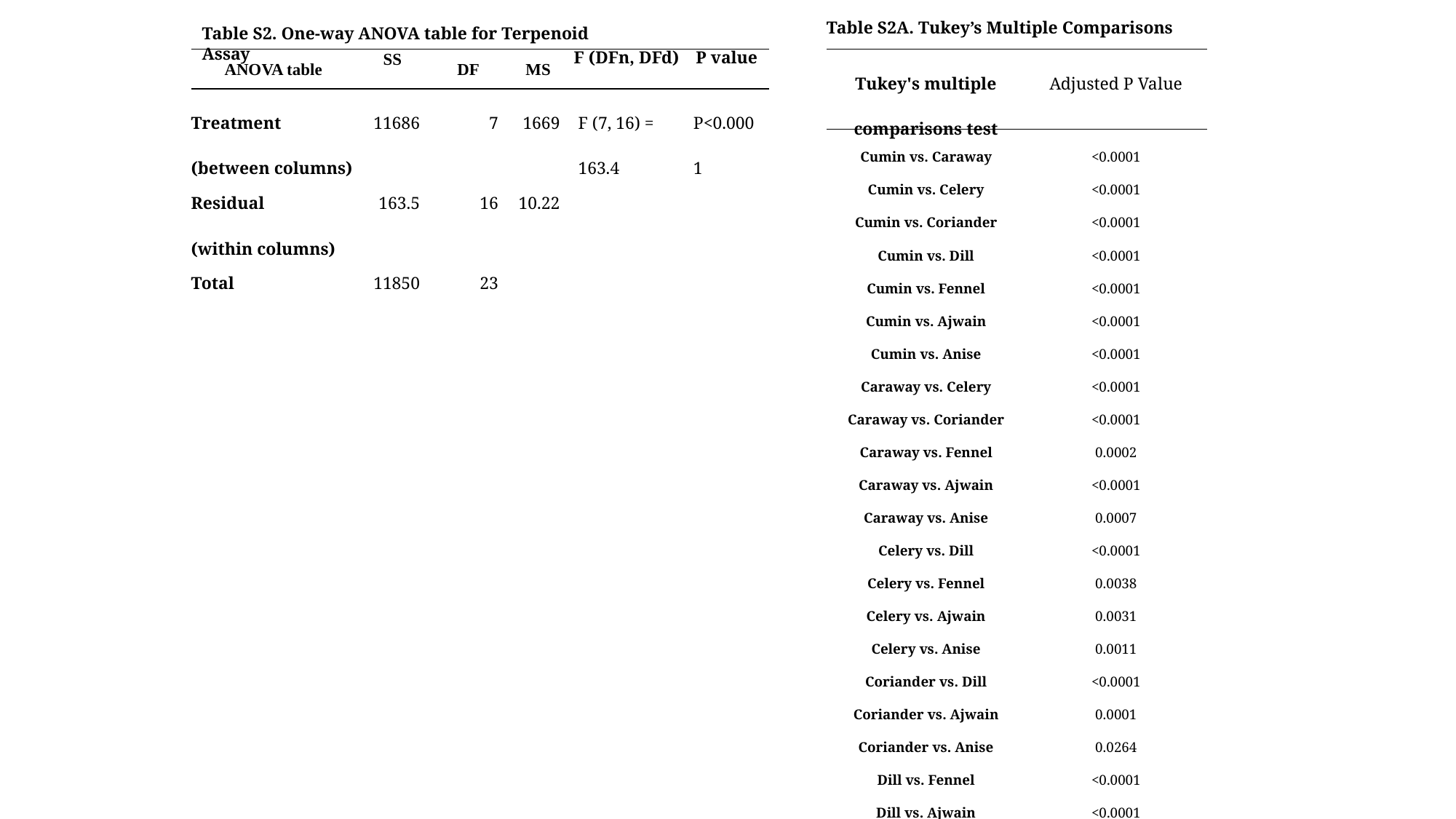

Table S2A. Tukey’s Multiple Comparisons
Table S2. One-way ANOVA table for Terpenoid Assay
| ANOVA table | SS | DF | MS | F (DFn, DFd) | P value |
| --- | --- | --- | --- | --- | --- |
| Treatment (between columns) | 11686 | 7 | 1669 | F (7, 16) = 163.4 | P<0.0001 |
| Residual (within columns) | 163.5 | 16 | 10.22 | | |
| Total | 11850 | 23 | | | |
| Tukey's multiple comparisons test | Adjusted P Value |
| --- | --- |
| Cumin vs. Caraway | <0.0001 |
| Cumin vs. Celery | <0.0001 |
| Cumin vs. Coriander | <0.0001 |
| Cumin vs. Dill | <0.0001 |
| Cumin vs. Fennel | <0.0001 |
| Cumin vs. Ajwain | <0.0001 |
| Cumin vs. Anise | <0.0001 |
| Caraway vs. Celery | <0.0001 |
| Caraway vs. Coriander | <0.0001 |
| Caraway vs. Fennel | 0.0002 |
| Caraway vs. Ajwain | <0.0001 |
| Caraway vs. Anise | 0.0007 |
| Celery vs. Dill | <0.0001 |
| Celery vs. Fennel | 0.0038 |
| Celery vs. Ajwain | 0.0031 |
| Celery vs. Anise | 0.0011 |
| Coriander vs. Dill | <0.0001 |
| Coriander vs. Ajwain | 0.0001 |
| Coriander vs. Anise | 0.0264 |
| Dill vs. Fennel | <0.0001 |
| Dill vs. Ajwain | <0.0001 |
| Dill vs. Anise | <0.0001 |
| Fennel vs. Ajwain | <0.0001 |
| Ajwain vs. Anise | <0.0001 |

## Slide 5
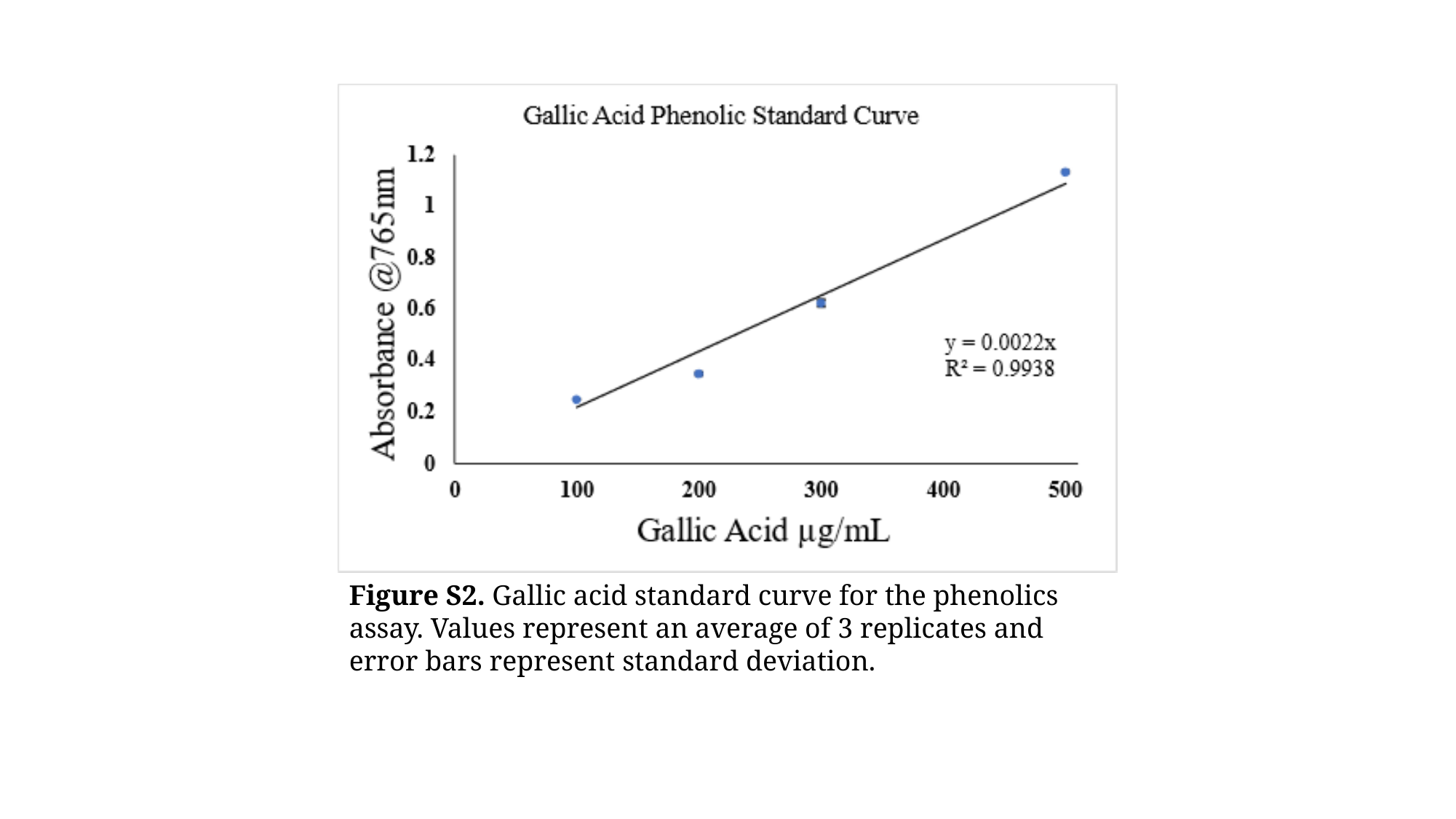

Figure S2. Gallic acid standard curve for the phenolics assay. Values represent an average of 3 replicates and error bars represent standard deviation.

## Slide 6
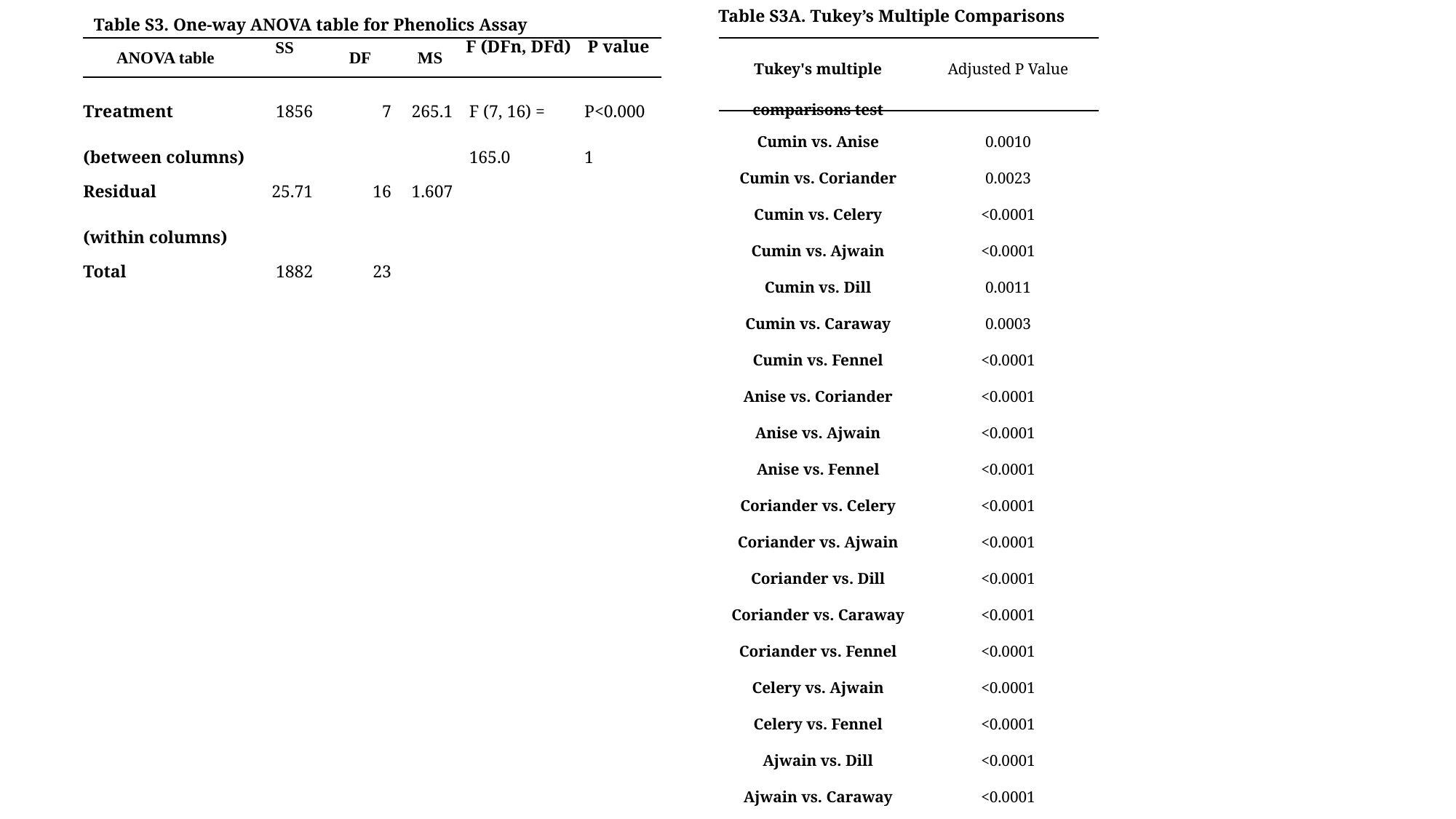

Table S3A. Tukey’s Multiple Comparisons
Table S3. One-way ANOVA table for Phenolics Assay
| ANOVA table | SS | DF | MS | F (DFn, DFd) | P value |
| --- | --- | --- | --- | --- | --- |
| Treatment (between columns) | 1856 | 7 | 265.1 | F (7, 16) = 165.0 | P<0.0001 |
| Residual (within columns) | 25.71 | 16 | 1.607 | | |
| Total | 1882 | 23 | | | |
| Tukey's multiple comparisons test | Adjusted P Value |
| --- | --- |
| Cumin vs. Anise | 0.0010 |
| Cumin vs. Coriander | 0.0023 |
| Cumin vs. Celery | <0.0001 |
| Cumin vs. Ajwain | <0.0001 |
| Cumin vs. Dill | 0.0011 |
| Cumin vs. Caraway | 0.0003 |
| Cumin vs. Fennel | <0.0001 |
| Anise vs. Coriander | <0.0001 |
| Anise vs. Ajwain | <0.0001 |
| Anise vs. Fennel | <0.0001 |
| Coriander vs. Celery | <0.0001 |
| Coriander vs. Ajwain | <0.0001 |
| Coriander vs. Dill | <0.0001 |
| Coriander vs. Caraway | <0.0001 |
| Coriander vs. Fennel | <0.0001 |
| Celery vs. Ajwain | <0.0001 |
| Celery vs. Fennel | <0.0001 |
| Ajwain vs. Dill | <0.0001 |
| Ajwain vs. Caraway | <0.0001 |
| Ajwain vs. Fennel | <0.0001 |
| Dill vs. Fennel | <0.0001 |
| Caraway vs. Fennel | <0.0001 |

## Slide 7
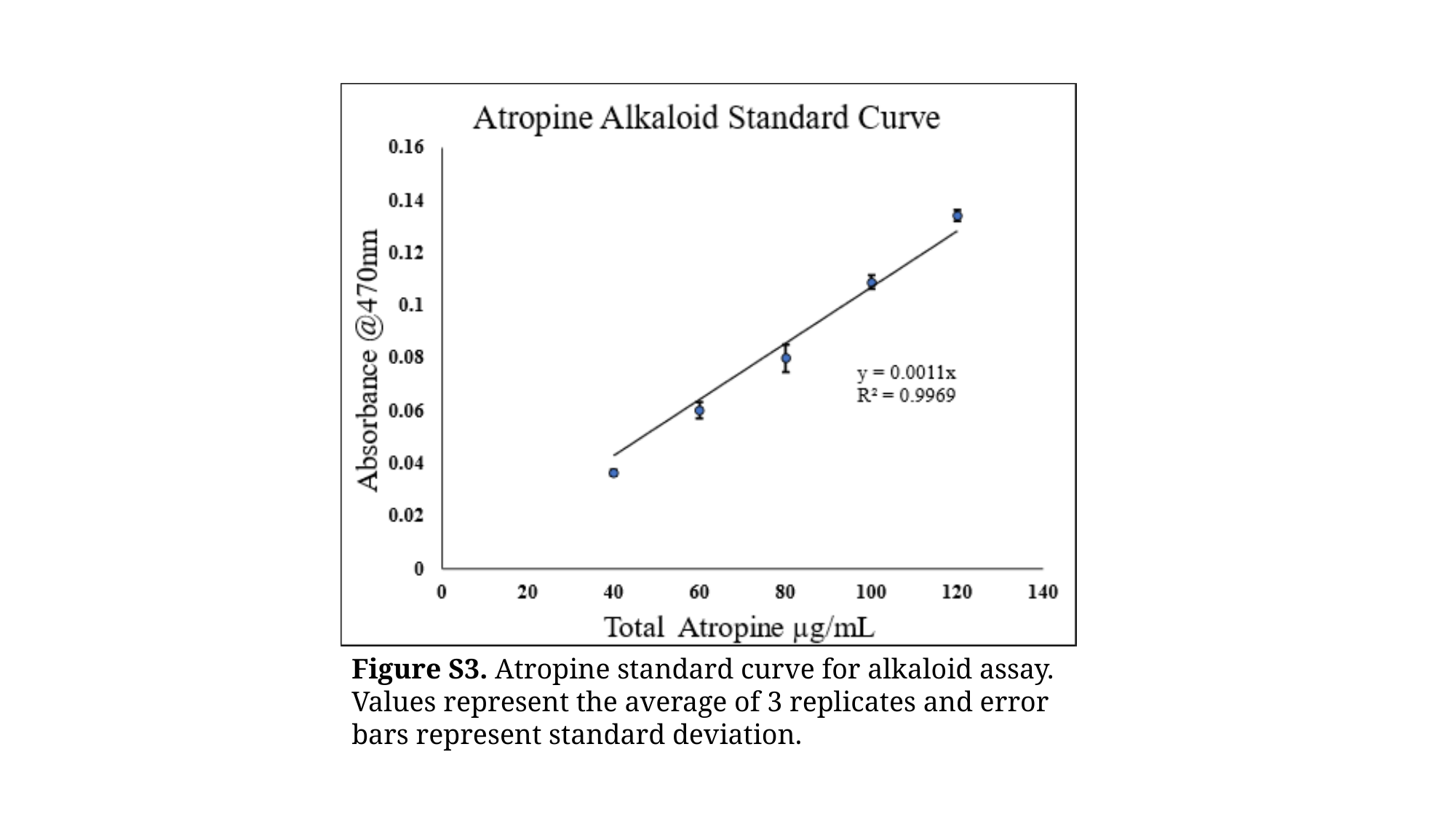

Figure S3. Atropine standard curve for alkaloid assay. Values represent the average of 3 replicates and error bars represent standard deviation.

## Slide 8
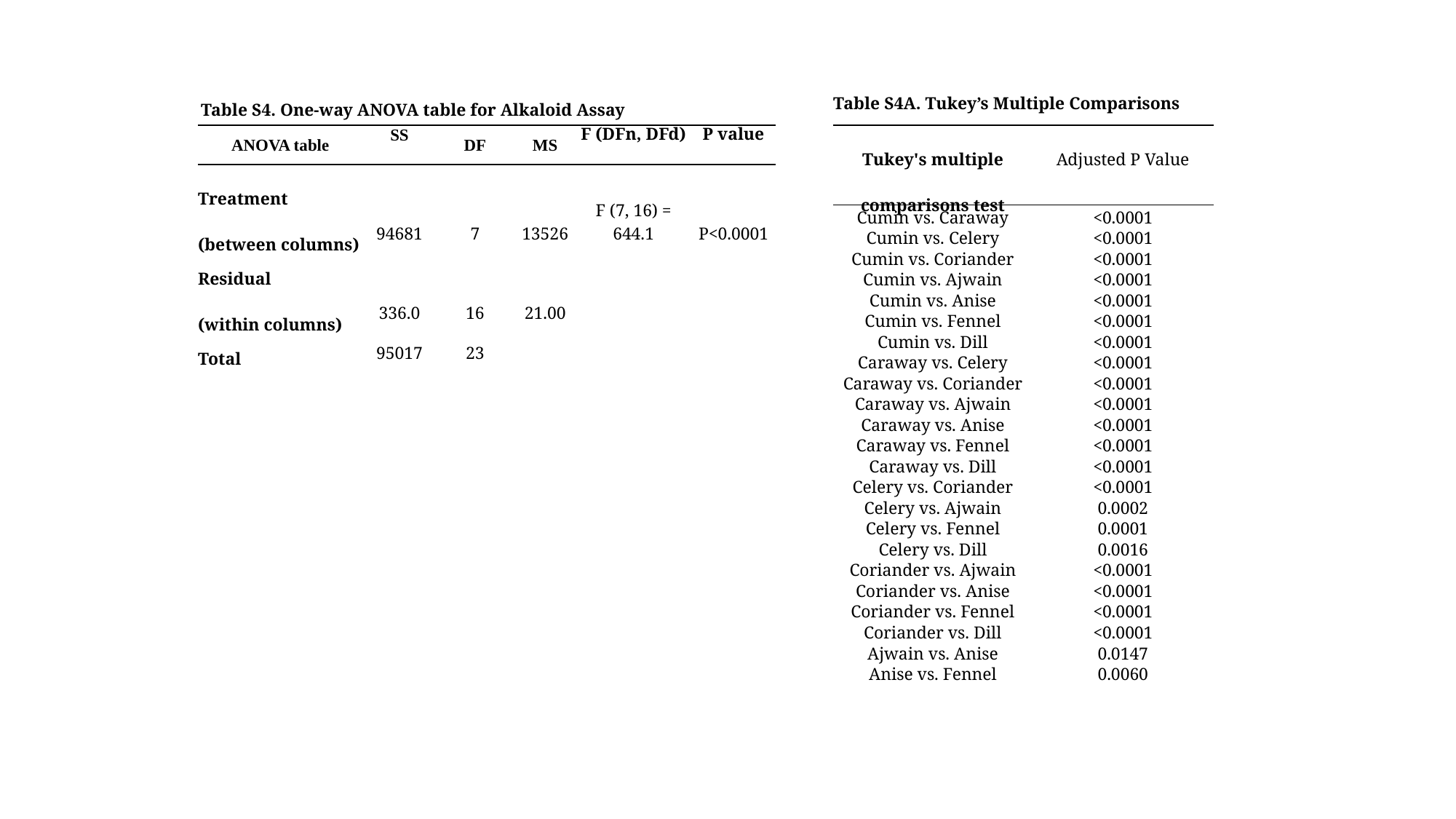

Table S4A. Tukey’s Multiple Comparisons
Table S4. One-way ANOVA table for Alkaloid Assay
| ANOVA table | SS | DF | MS | F (DFn, DFd) | P value |
| --- | --- | --- | --- | --- | --- |
| Treatment (between columns) | 94681 | 7 | 13526 | F (7, 16) = 644.1 | P<0.0001 |
| Residual (within columns) | 336.0 | 16 | 21.00 | | |
| Total | 95017 | 23 | | | |
| Tukey's multiple comparisons test | Adjusted P Value |
| --- | --- |
| Cumin vs. Caraway | <0.0001 |
| Cumin vs. Celery | <0.0001 |
| Cumin vs. Coriander | <0.0001 |
| Cumin vs. Ajwain | <0.0001 |
| Cumin vs. Anise | <0.0001 |
| Cumin vs. Fennel | <0.0001 |
| Cumin vs. Dill | <0.0001 |
| Caraway vs. Celery | <0.0001 |
| Caraway vs. Coriander | <0.0001 |
| Caraway vs. Ajwain | <0.0001 |
| Caraway vs. Anise | <0.0001 |
| Caraway vs. Fennel | <0.0001 |
| Caraway vs. Dill | <0.0001 |
| Celery vs. Coriander | <0.0001 |
| Celery vs. Ajwain | 0.0002 |
| Celery vs. Fennel | 0.0001 |
| Celery vs. Dill | 0.0016 |
| Coriander vs. Ajwain | <0.0001 |
| Coriander vs. Anise | <0.0001 |
| Coriander vs. Fennel | <0.0001 |
| Coriander vs. Dill | <0.0001 |
| Ajwain vs. Anise | 0.0147 |
| Anise vs. Fennel | 0.0060 |

## Slide 9
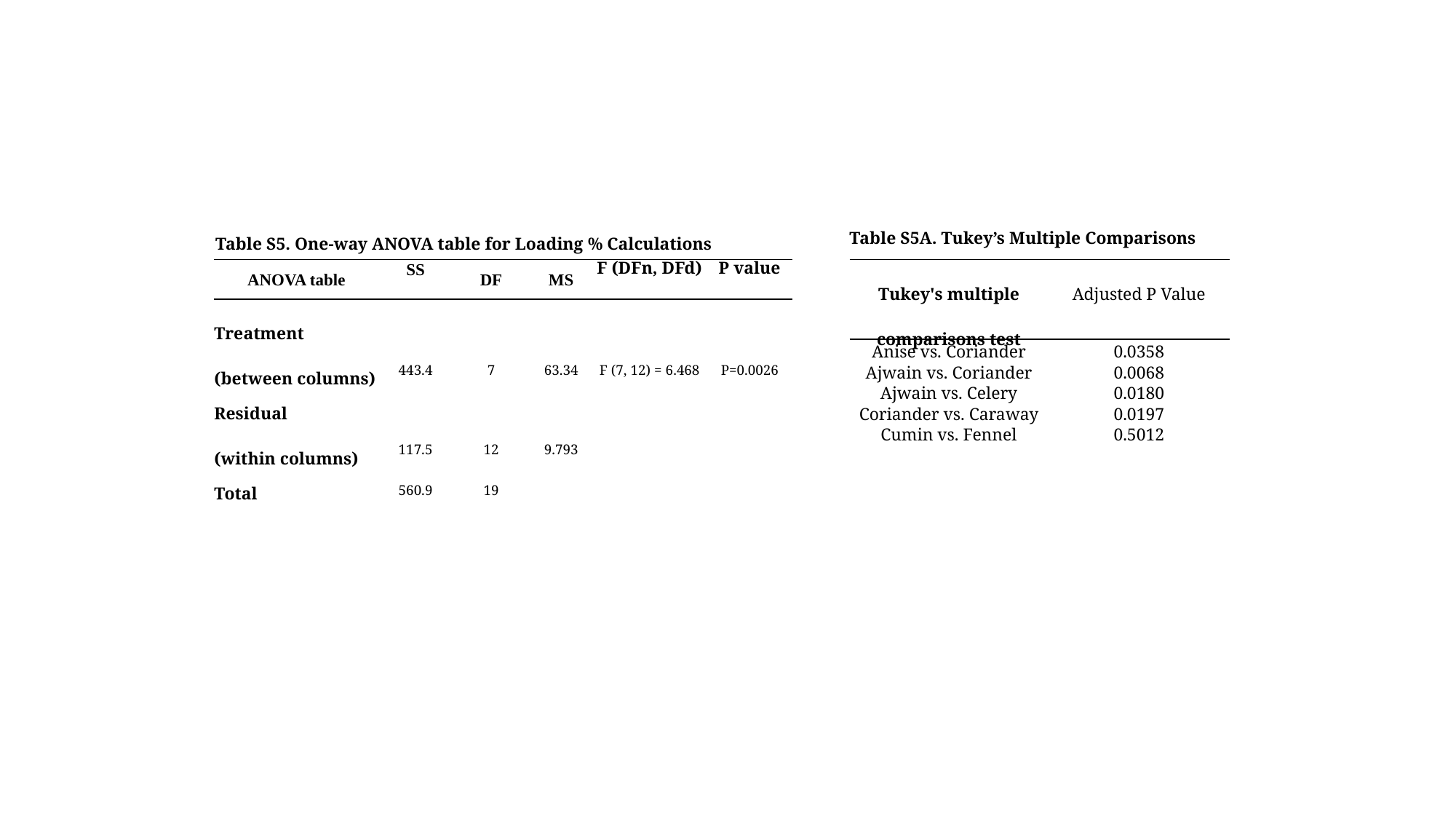

Table S5A. Tukey’s Multiple Comparisons
Table S5. One-way ANOVA table for Loading % Calculations
| ANOVA table | SS | DF | MS | F (DFn, DFd) | P value |
| --- | --- | --- | --- | --- | --- |
| Treatment (between columns) | 443.4 | 7 | 63.34 | F (7, 12) = 6.468 | P=0.0026 |
| Residual (within columns) | 117.5 | 12 | 9.793 | | |
| Total | 560.9 | 19 | | | |
| Tukey's multiple comparisons test | Adjusted P Value |
| --- | --- |
| Anise vs. Coriander | 0.0358 |
| Ajwain vs. Coriander | 0.0068 |
| Ajwain vs. Celery | 0.0180 |
| Coriander vs. Caraway | 0.0197 |
| Cumin vs. Fennel | 0.5012 |

## Slide 10
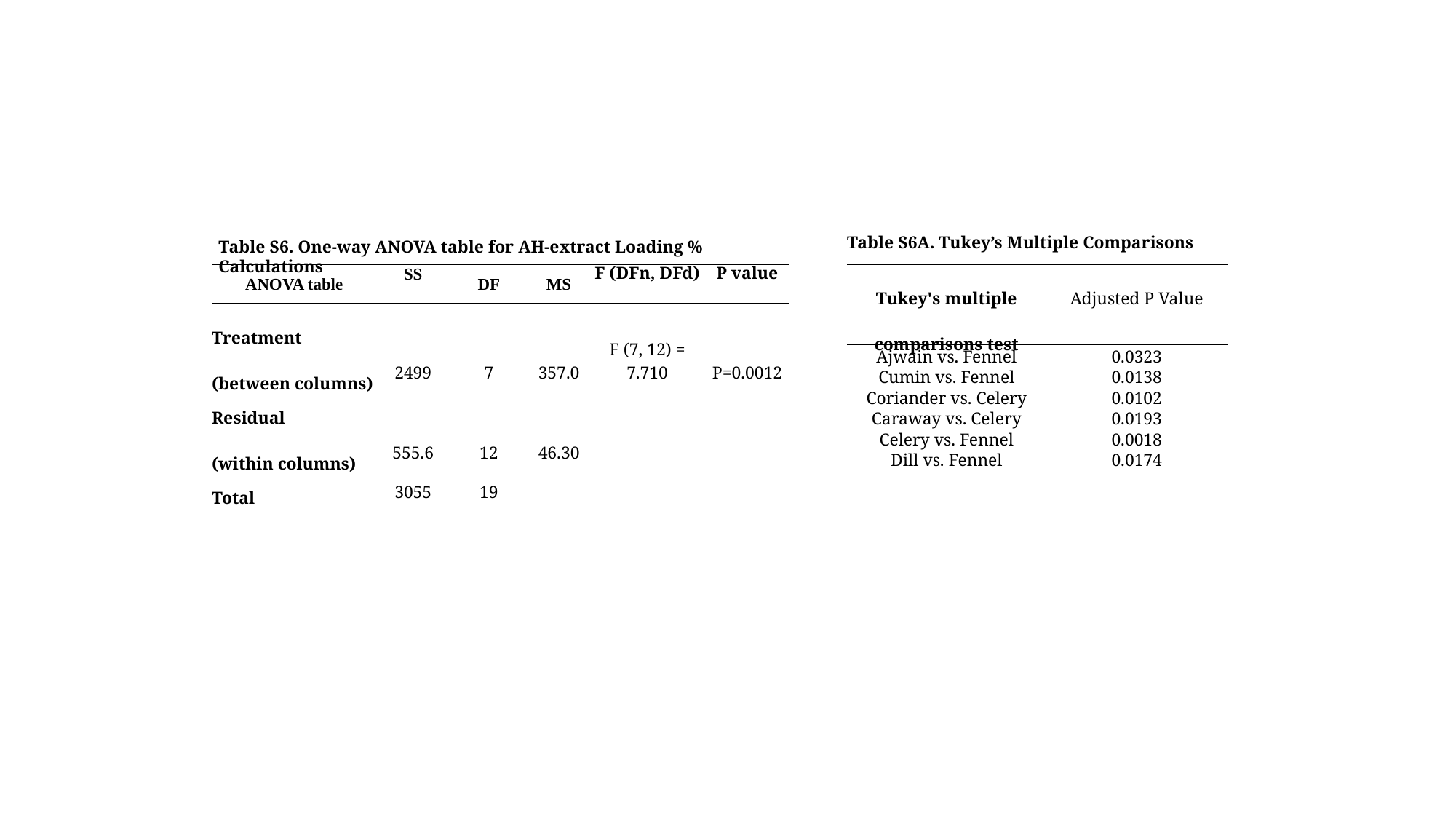

Table S6A. Tukey’s Multiple Comparisons
Table S6. One-way ANOVA table for AH-extract Loading % Calculations
| ANOVA table | SS | DF | MS | F (DFn, DFd) | P value |
| --- | --- | --- | --- | --- | --- |
| Treatment (between columns) | 2499 | 7 | 357.0 | F (7, 12) = 7.710 | P=0.0012 |
| Residual (within columns) | 555.6 | 12 | 46.30 | | |
| Total | 3055 | 19 | | | |
| Tukey's multiple comparisons test | Adjusted P Value |
| --- | --- |
| Ajwain vs. Fennel | 0.0323 |
| Cumin vs. Fennel | 0.0138 |
| Coriander vs. Celery | 0.0102 |
| Caraway vs. Celery | 0.0193 |
| Celery vs. Fennel | 0.0018 |
| Dill vs. Fennel | 0.0174 |

## Slide 11
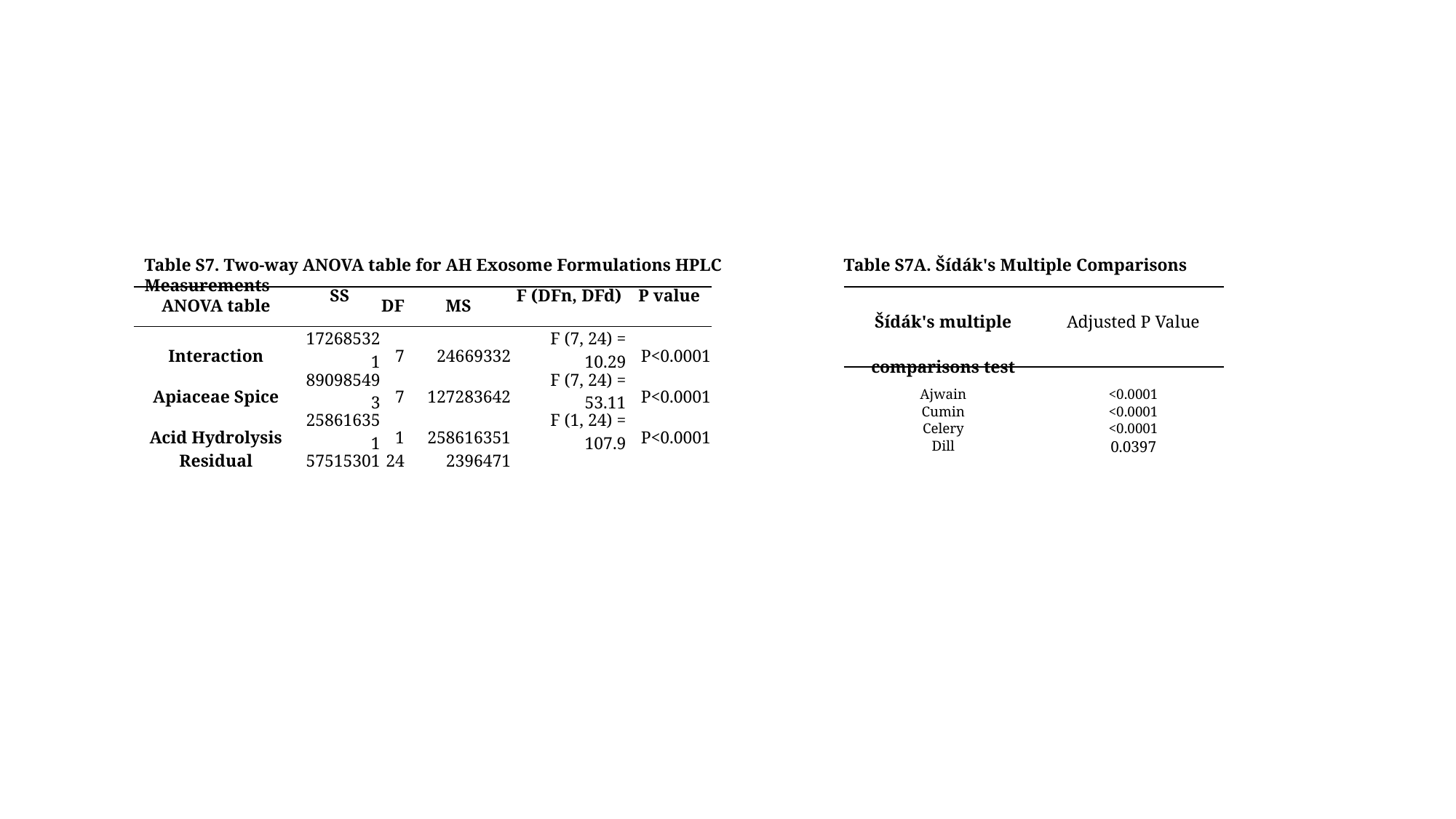

Table S7. Two-way ANOVA table for AH Exosome Formulations HPLC Measurements
Table S7A. Šídák's Multiple Comparisons
| ANOVA table | SS | DF | MS | F (DFn, DFd) | P value |
| --- | --- | --- | --- | --- | --- |
| Interaction | 172685321 | 7 | 24669332 | F (7, 24) = 10.29 | P<0.0001 |
| Apiaceae Spice | 890985493 | 7 | 127283642 | F (7, 24) = 53.11 | P<0.0001 |
| Acid Hydrolysis | 258616351 | 1 | 258616351 | F (1, 24) = 107.9 | P<0.0001 |
| Residual | 57515301 | 24 | 2396471 | | |
| Šídák's multiple comparisons test | Adjusted P Value |
| --- | --- |
| | |
| Ajwain | <0.0001 |
| Cumin | <0.0001 |
| Celery | <0.0001 |
| Dill | 0.0397 |

## Slide 12
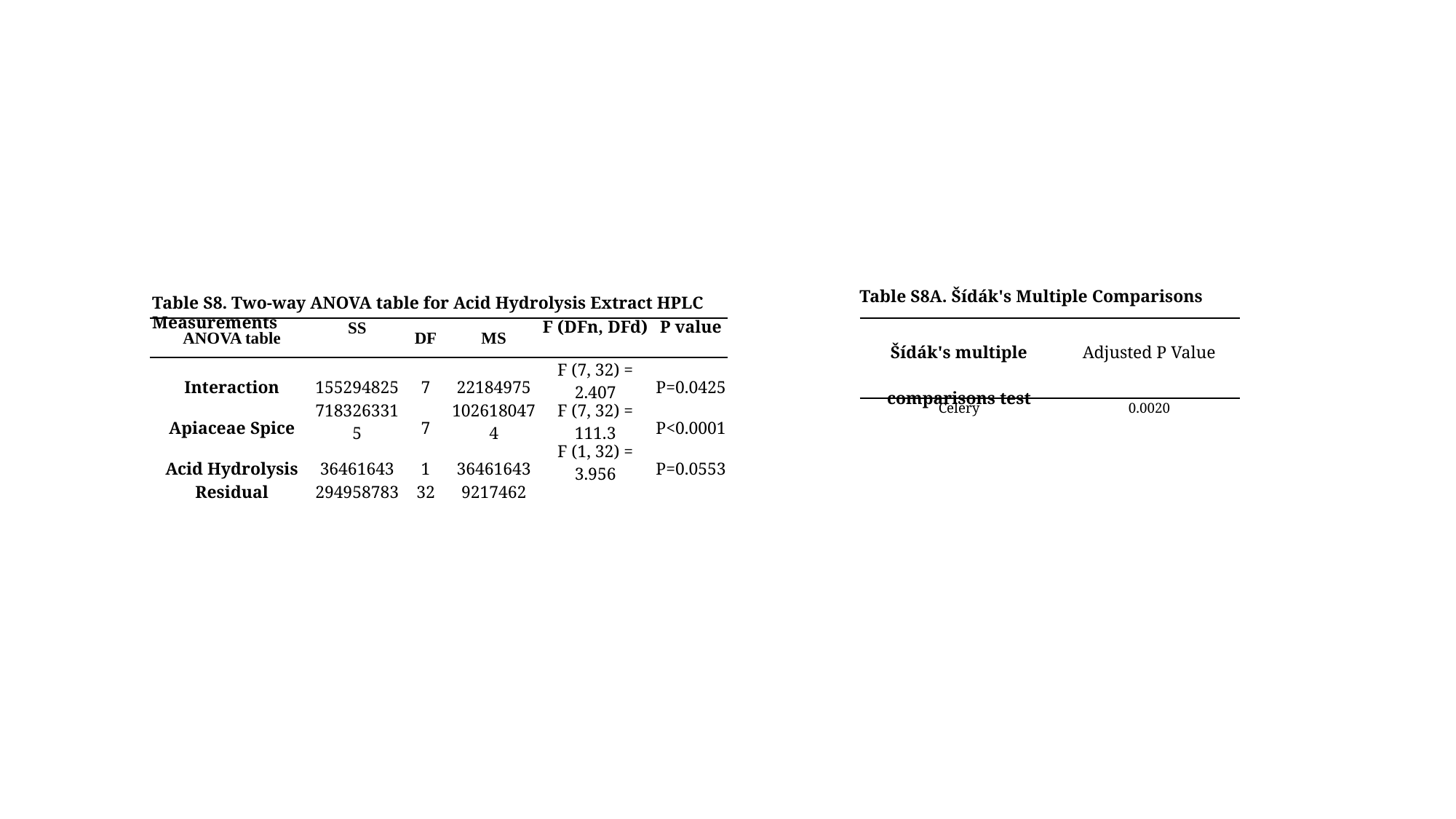

Table S8A. Šídák's Multiple Comparisons
Table S8. Two-way ANOVA table for Acid Hydrolysis Extract HPLC Measurements
| ANOVA table | SS | DF | MS | F (DFn, DFd) | P value |
| --- | --- | --- | --- | --- | --- |
| Interaction | 155294825 | 7 | 22184975 | F (7, 32) = 2.407 | P=0.0425 |
| Apiaceae Spice | 7183263315 | 7 | 1026180474 | F (7, 32) = 111.3 | P<0.0001 |
| Acid Hydrolysis | 36461643 | 1 | 36461643 | F (1, 32) = 3.956 | P=0.0553 |
| Residual | 294958783 | 32 | 9217462 | | |
| Šídák's multiple comparisons test | Adjusted P Value |
| --- | --- |
| Celery | 0.0020 |

## Slide 13
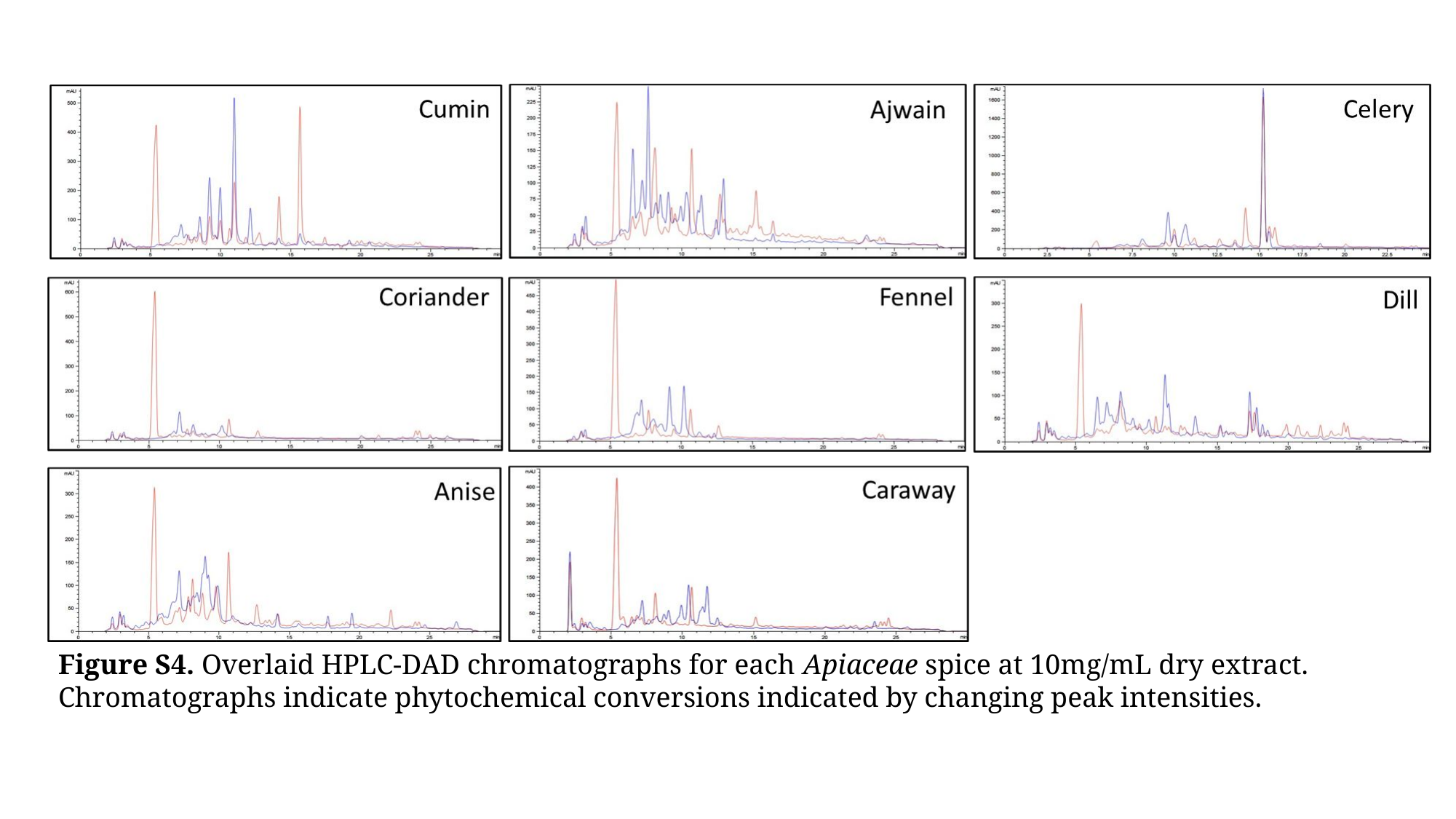

Figure S4. Overlaid HPLC-DAD chromatographs for each Apiaceae spice at 10mg/mL dry extract. Chromatographs indicate phytochemical conversions indicated by changing peak intensities.

## Slide 14
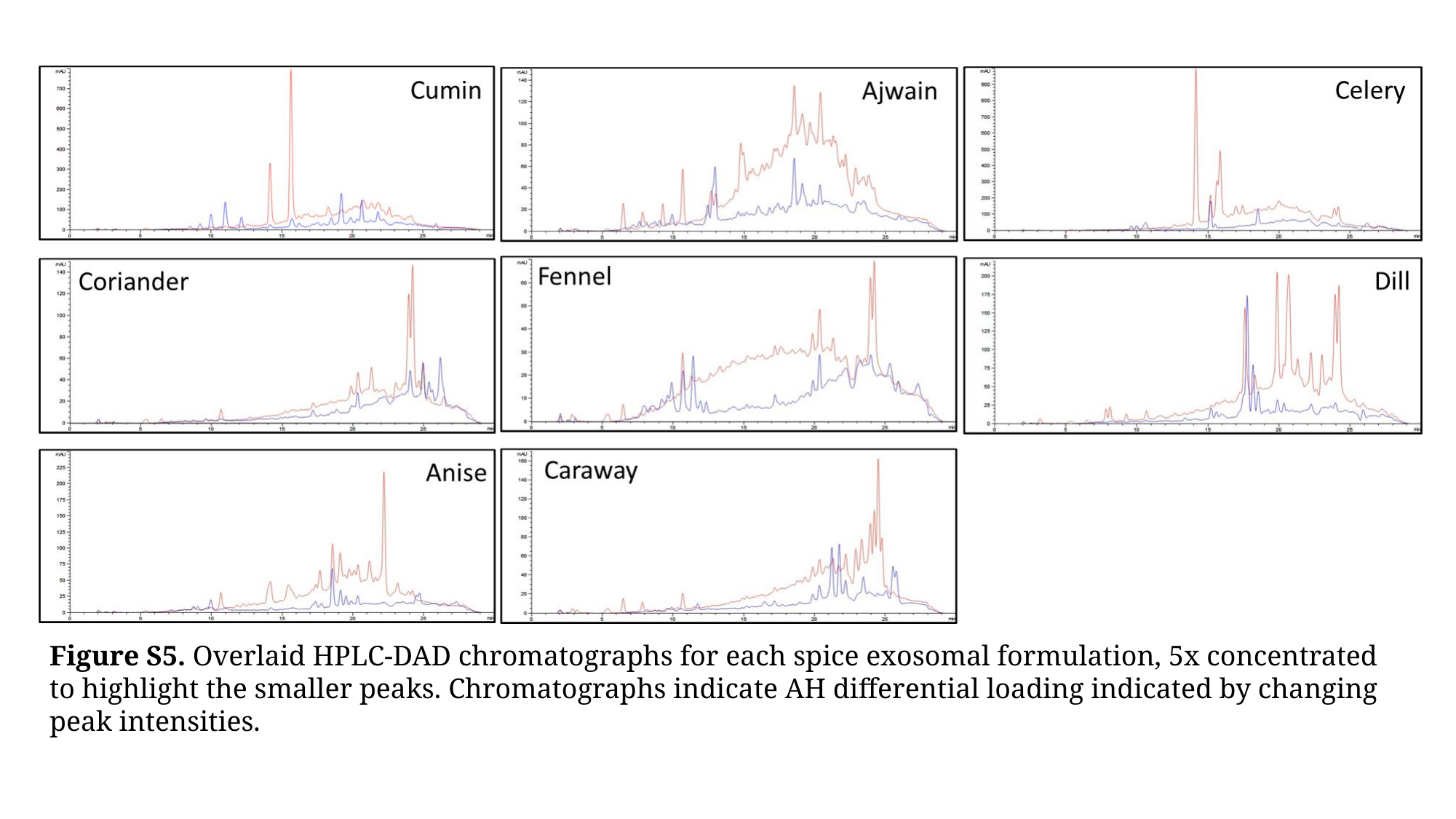

Figure S5. Overlaid HPLC-DAD chromatographs for each spice exosomal formulation, 5x concentrated to highlight the smaller peaks. Chromatographs indicate AH differential loading indicated by changing peak intensities.

## Slide 15
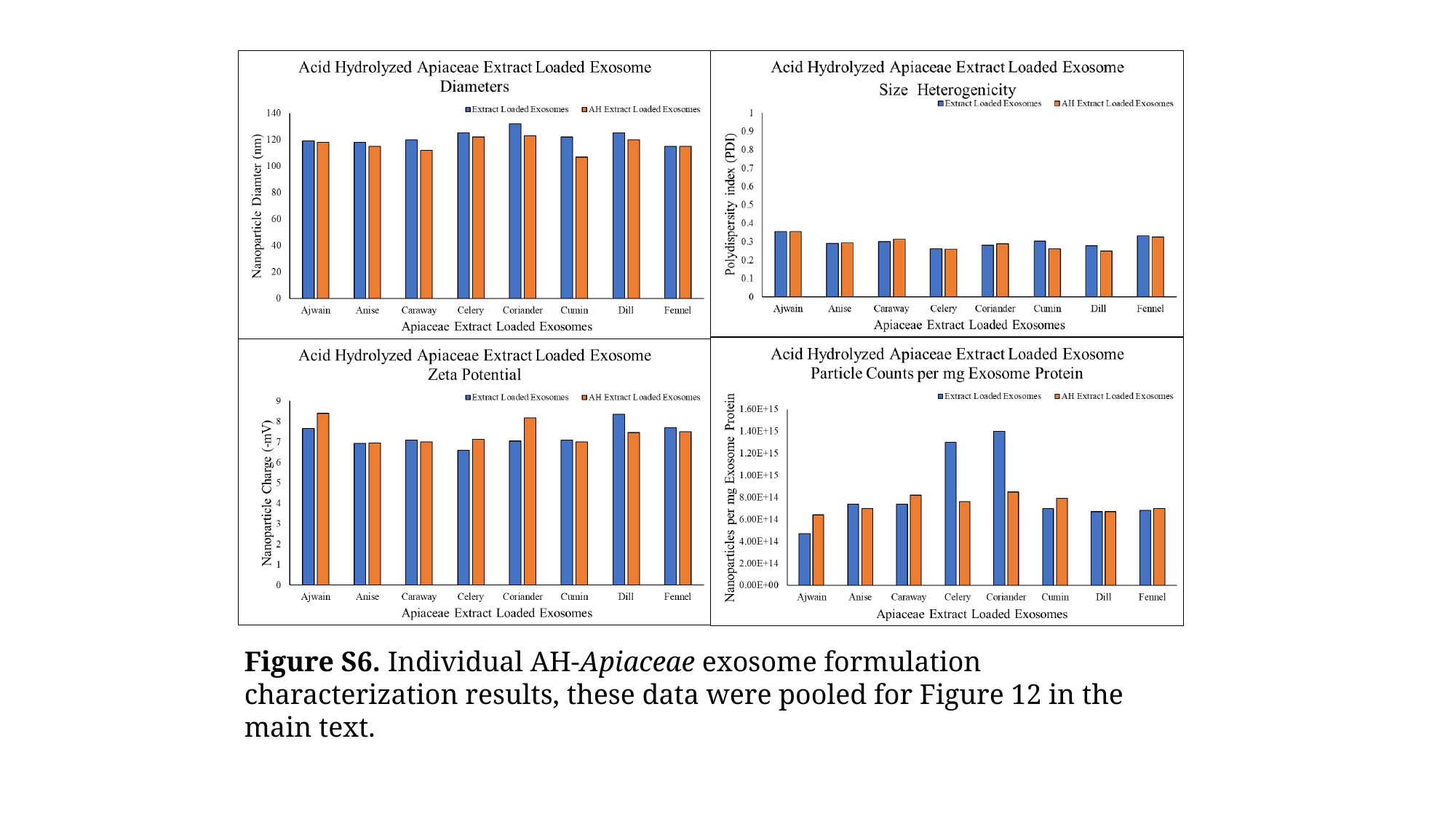

Figure S6. Individual AH-Apiaceae exosome formulation characterization results, these data were pooled for Figure 12 in the main text.

## Slide 16
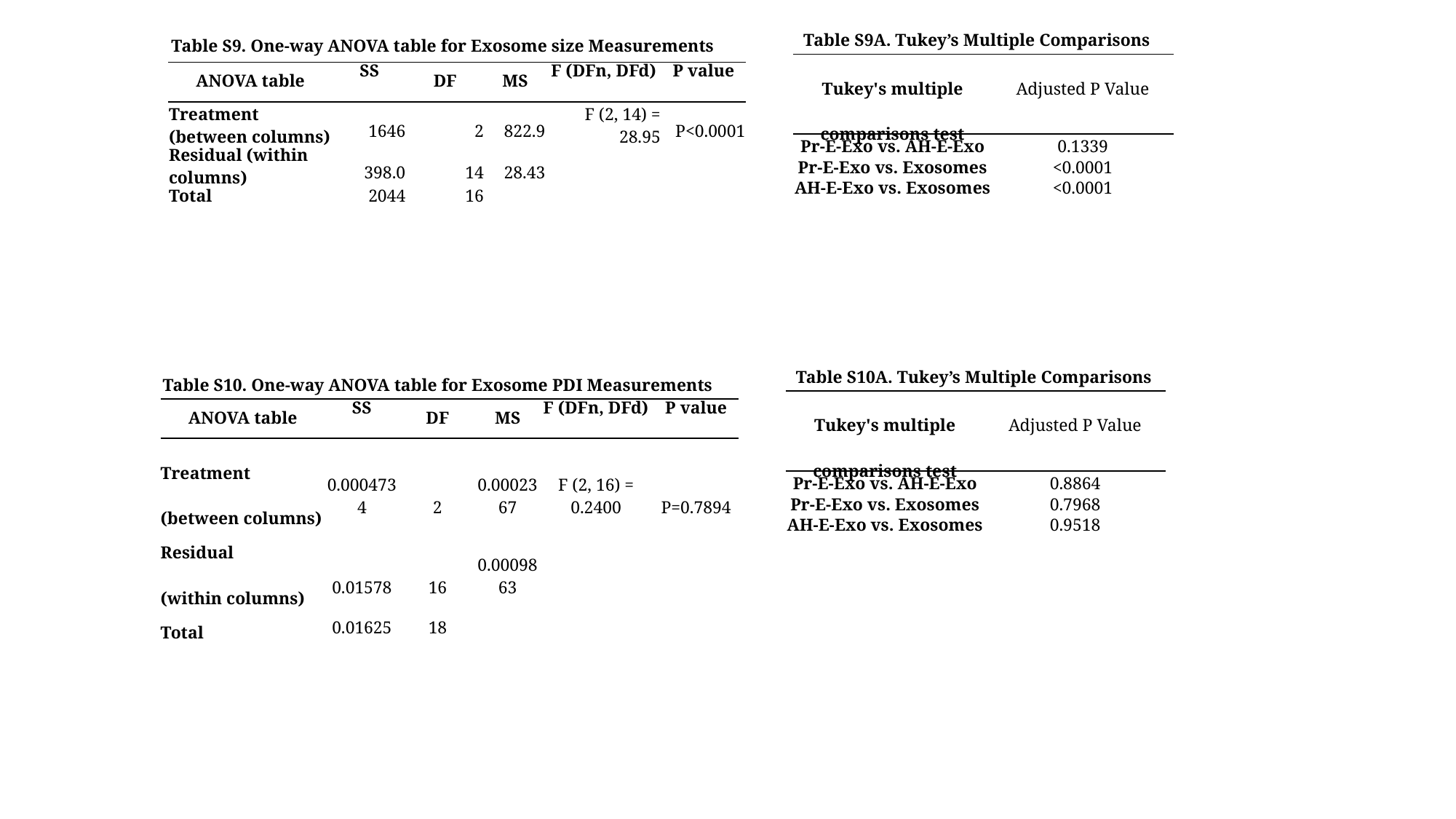

Table S9A. Tukey’s Multiple Comparisons
Table S9. One-way ANOVA table for Exosome size Measurements
| Tukey's multiple comparisons test | Adjusted P Value |
| --- | --- |
| Pr-E-Exo vs. AH-E-Exo | 0.1339 |
| Pr-E-Exo vs. Exosomes | <0.0001 |
| AH-E-Exo vs. Exosomes | <0.0001 |
| ANOVA table | SS | DF | MS | F (DFn, DFd) | P value |
| --- | --- | --- | --- | --- | --- |
| Treatment (between columns) | 1646 | 2 | 822.9 | F (2, 14) = 28.95 | P<0.0001 |
| Residual (within columns) | 398.0 | 14 | 28.43 | | |
| Total | 2044 | 16 | | | |
Table S10A. Tukey’s Multiple Comparisons
Table S10. One-way ANOVA table for Exosome PDI Measurements
| Tukey's multiple comparisons test | Adjusted P Value |
| --- | --- |
| Pr-E-Exo vs. AH-E-Exo | 0.8864 |
| Pr-E-Exo vs. Exosomes | 0.7968 |
| AH-E-Exo vs. Exosomes | 0.9518 |
| ANOVA table | SS | DF | MS | F (DFn, DFd) | P value |
| --- | --- | --- | --- | --- | --- |
| Treatment (between columns) | 0.0004734 | 2 | 0.0002367 | F (2, 16) = 0.2400 | P=0.7894 |
| Residual (within columns) | 0.01578 | 16 | 0.0009863 | | |
| Total | 0.01625 | 18 | | | |

## Slide 17
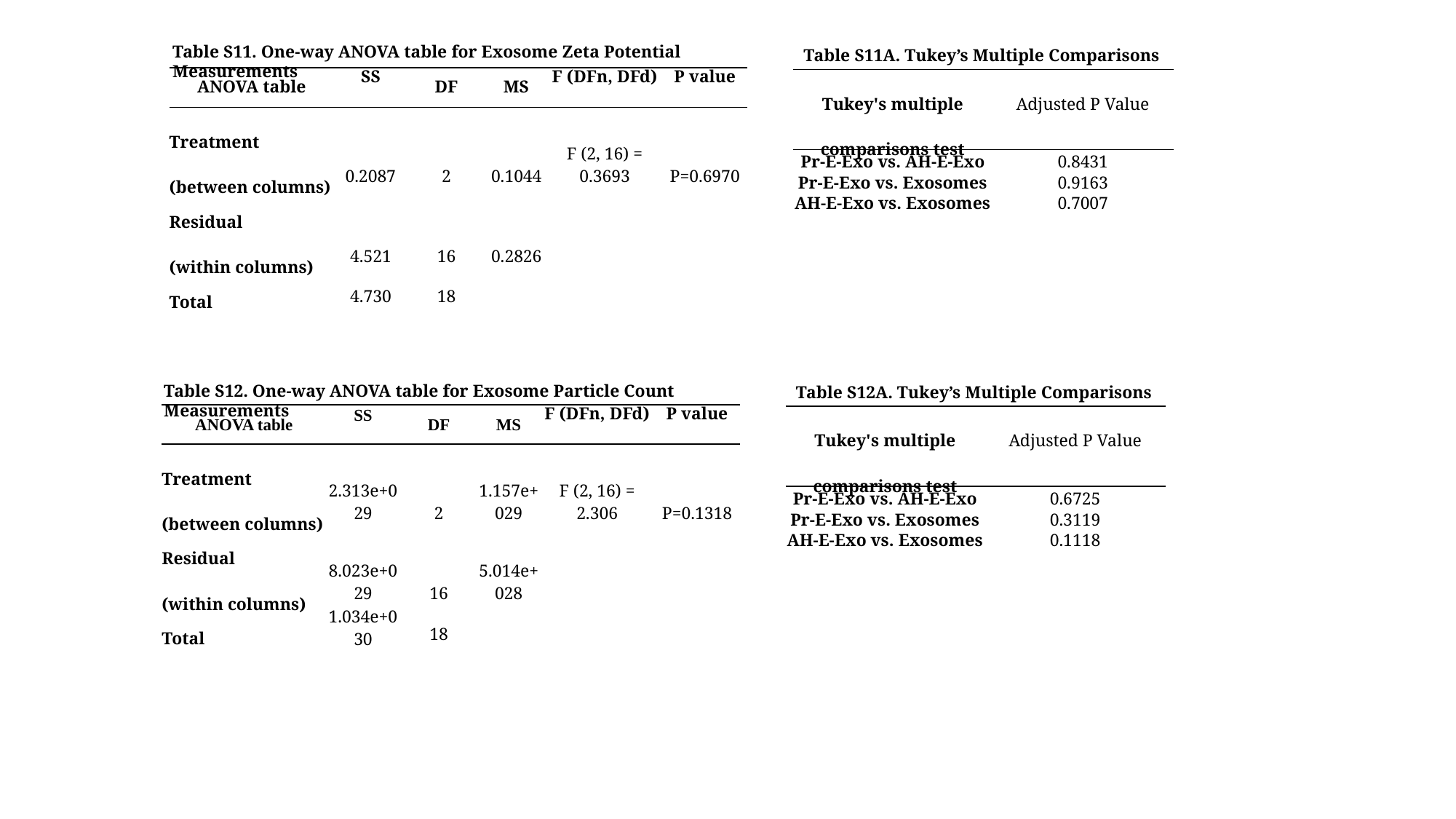

Table S11. One-way ANOVA table for Exosome Zeta Potential Measurements
Table S11A. Tukey’s Multiple Comparisons
| ANOVA table | SS | DF | MS | F (DFn, DFd) | P value |
| --- | --- | --- | --- | --- | --- |
| Treatment (between columns) | 0.2087 | 2 | 0.1044 | F (2, 16) = 0.3693 | P=0.6970 |
| Residual (within columns) | 4.521 | 16 | 0.2826 | | |
| Total | 4.730 | 18 | | | |
| Tukey's multiple comparisons test | Adjusted P Value |
| --- | --- |
| Pr-E-Exo vs. AH-E-Exo | 0.8431 |
| Pr-E-Exo vs. Exosomes | 0.9163 |
| AH-E-Exo vs. Exosomes | 0.7007 |
Table S12. One-way ANOVA table for Exosome Particle Count Measurements
Table S12A. Tukey’s Multiple Comparisons
| ANOVA table | SS | DF | MS | F (DFn, DFd) | P value |
| --- | --- | --- | --- | --- | --- |
| Treatment (between columns) | 2.313e+029 | 2 | 1.157e+029 | F (2, 16) = 2.306 | P=0.1318 |
| Residual (within columns) | 8.023e+029 | 16 | 5.014e+028 | | |
| Total | 1.034e+030 | 18 | | | |
| Tukey's multiple comparisons test | Adjusted P Value |
| --- | --- |
| Pr-E-Exo vs. AH-E-Exo | 0.6725 |
| Pr-E-Exo vs. Exosomes | 0.3119 |
| AH-E-Exo vs. Exosomes | 0.1118 |

## Slide 18
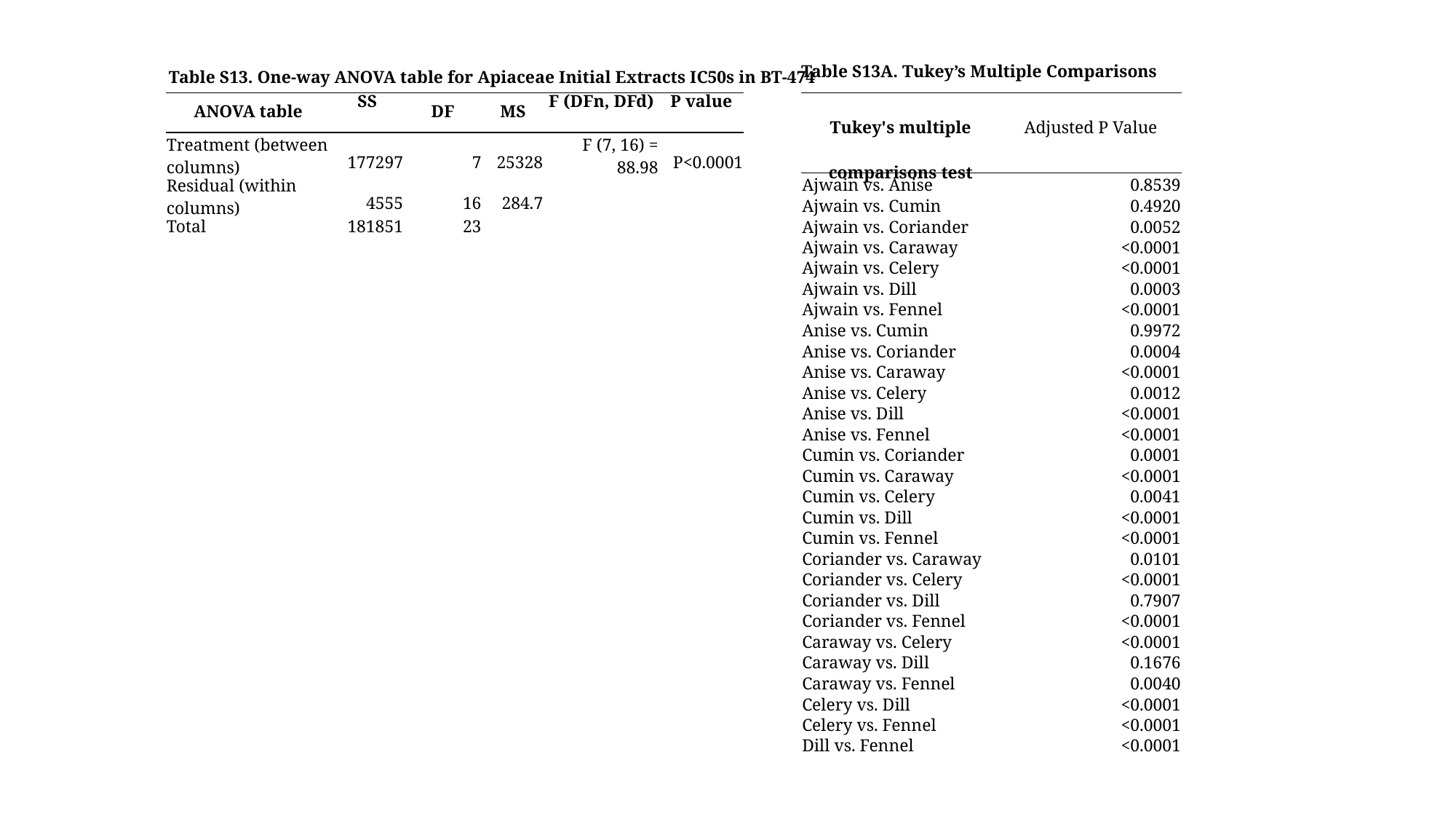

Table S13A. Tukey’s Multiple Comparisons
Table S13. One-way ANOVA table for Apiaceae Initial Extracts IC50s in BT-474
| ANOVA table | SS | DF | MS | F (DFn, DFd) | P value |
| --- | --- | --- | --- | --- | --- |
| Treatment (between columns) | 177297 | 7 | 25328 | F (7, 16) = 88.98 | P<0.0001 |
| Residual (within columns) | 4555 | 16 | 284.7 | | |
| Total | 181851 | 23 | | | |
| Tukey's multiple comparisons test | Adjusted P Value |
| --- | --- |
| Ajwain vs. Anise | 0.8539 |
| Ajwain vs. Cumin | 0.4920 |
| Ajwain vs. Coriander | 0.0052 |
| Ajwain vs. Caraway | <0.0001 |
| Ajwain vs. Celery | <0.0001 |
| Ajwain vs. Dill | 0.0003 |
| Ajwain vs. Fennel | <0.0001 |
| Anise vs. Cumin | 0.9972 |
| Anise vs. Coriander | 0.0004 |
| Anise vs. Caraway | <0.0001 |
| Anise vs. Celery | 0.0012 |
| Anise vs. Dill | <0.0001 |
| Anise vs. Fennel | <0.0001 |
| Cumin vs. Coriander | 0.0001 |
| Cumin vs. Caraway | <0.0001 |
| Cumin vs. Celery | 0.0041 |
| Cumin vs. Dill | <0.0001 |
| Cumin vs. Fennel | <0.0001 |
| Coriander vs. Caraway | 0.0101 |
| Coriander vs. Celery | <0.0001 |
| Coriander vs. Dill | 0.7907 |
| Coriander vs. Fennel | <0.0001 |
| Caraway vs. Celery | <0.0001 |
| Caraway vs. Dill | 0.1676 |
| Caraway vs. Fennel | 0.0040 |
| Celery vs. Dill | <0.0001 |
| Celery vs. Fennel | <0.0001 |
| Dill vs. Fennel | <0.0001 |

## Slide 19
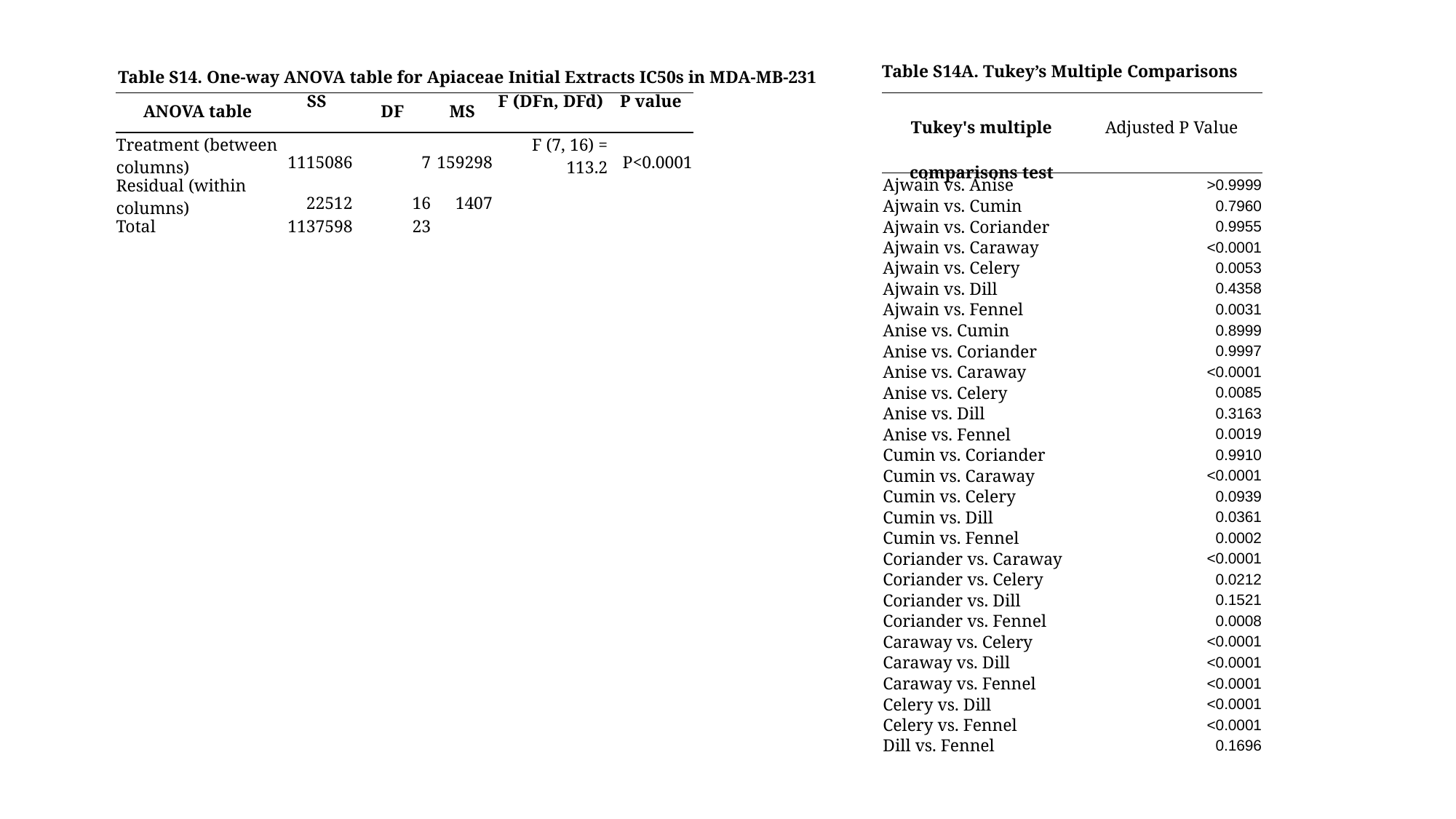

Table S14A. Tukey’s Multiple Comparisons
Table S14. One-way ANOVA table for Apiaceae Initial Extracts IC50s in MDA-MB-231
| ANOVA table | SS | DF | MS | F (DFn, DFd) | P value |
| --- | --- | --- | --- | --- | --- |
| Treatment (between columns) | 1115086 | 7 | 159298 | F (7, 16) = 113.2 | P<0.0001 |
| Residual (within columns) | 22512 | 16 | 1407 | | |
| Total | 1137598 | 23 | | | |
| Tukey's multiple comparisons test | Adjusted P Value |
| --- | --- |
| Ajwain vs. Anise | >0.9999 |
| Ajwain vs. Cumin | 0.7960 |
| Ajwain vs. Coriander | 0.9955 |
| Ajwain vs. Caraway | <0.0001 |
| Ajwain vs. Celery | 0.0053 |
| Ajwain vs. Dill | 0.4358 |
| Ajwain vs. Fennel | 0.0031 |
| Anise vs. Cumin | 0.8999 |
| Anise vs. Coriander | 0.9997 |
| Anise vs. Caraway | <0.0001 |
| Anise vs. Celery | 0.0085 |
| Anise vs. Dill | 0.3163 |
| Anise vs. Fennel | 0.0019 |
| Cumin vs. Coriander | 0.9910 |
| Cumin vs. Caraway | <0.0001 |
| Cumin vs. Celery | 0.0939 |
| Cumin vs. Dill | 0.0361 |
| Cumin vs. Fennel | 0.0002 |
| Coriander vs. Caraway | <0.0001 |
| Coriander vs. Celery | 0.0212 |
| Coriander vs. Dill | 0.1521 |
| Coriander vs. Fennel | 0.0008 |
| Caraway vs. Celery | <0.0001 |
| Caraway vs. Dill | <0.0001 |
| Caraway vs. Fennel | <0.0001 |
| Celery vs. Dill | <0.0001 |
| Celery vs. Fennel | <0.0001 |
| Dill vs. Fennel | 0.1696 |

## Slide 20
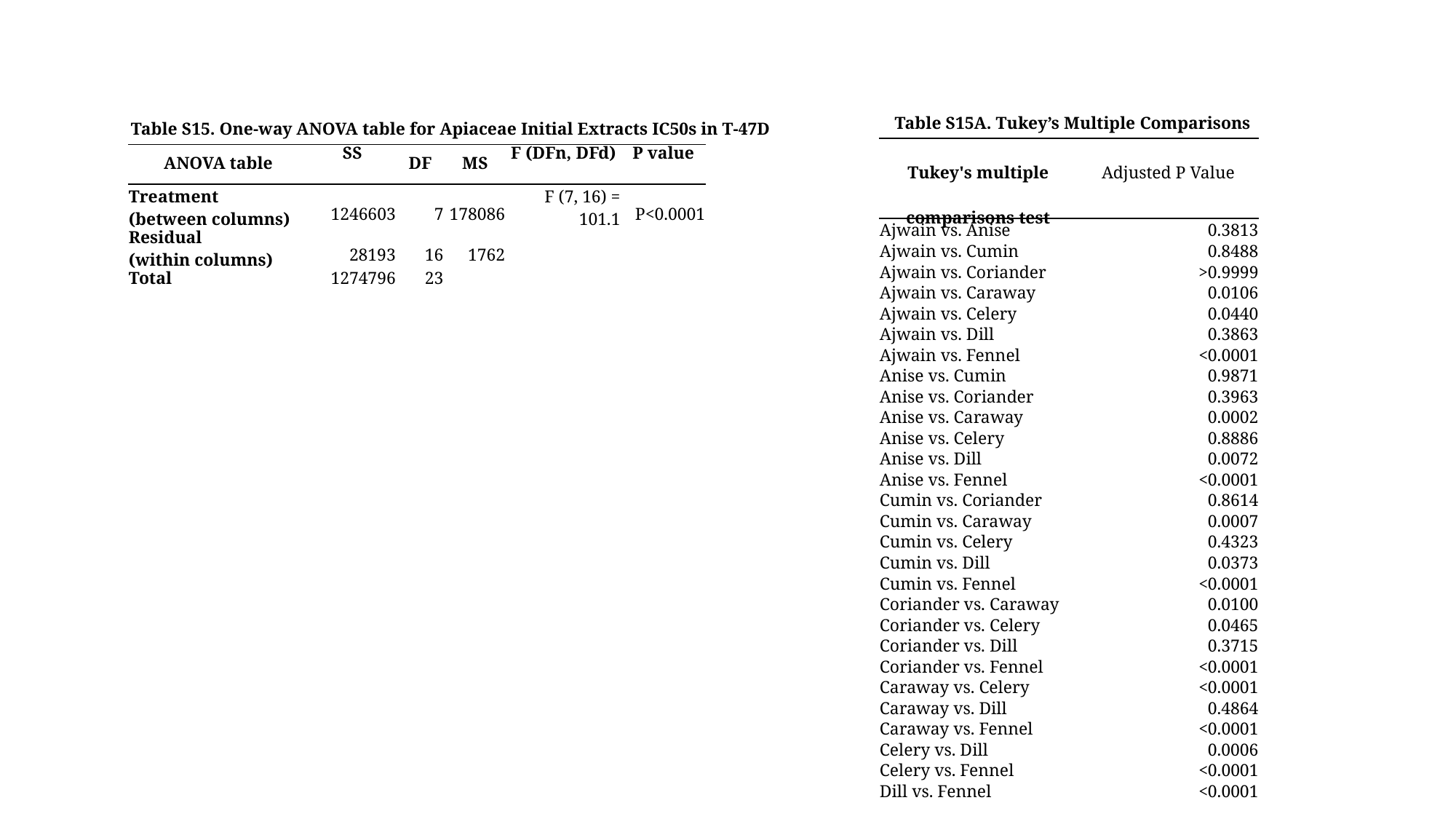

Table S15A. Tukey’s Multiple Comparisons
Table S15. One-way ANOVA table for Apiaceae Initial Extracts IC50s in T-47D
| Tukey's multiple comparisons test | Adjusted P Value |
| --- | --- |
| Ajwain vs. Anise | 0.3813 |
| Ajwain vs. Cumin | 0.8488 |
| Ajwain vs. Coriander | >0.9999 |
| Ajwain vs. Caraway | 0.0106 |
| Ajwain vs. Celery | 0.0440 |
| Ajwain vs. Dill | 0.3863 |
| Ajwain vs. Fennel | <0.0001 |
| Anise vs. Cumin | 0.9871 |
| Anise vs. Coriander | 0.3963 |
| Anise vs. Caraway | 0.0002 |
| Anise vs. Celery | 0.8886 |
| Anise vs. Dill | 0.0072 |
| Anise vs. Fennel | <0.0001 |
| Cumin vs. Coriander | 0.8614 |
| Cumin vs. Caraway | 0.0007 |
| Cumin vs. Celery | 0.4323 |
| Cumin vs. Dill | 0.0373 |
| Cumin vs. Fennel | <0.0001 |
| Coriander vs. Caraway | 0.0100 |
| Coriander vs. Celery | 0.0465 |
| Coriander vs. Dill | 0.3715 |
| Coriander vs. Fennel | <0.0001 |
| Caraway vs. Celery | <0.0001 |
| Caraway vs. Dill | 0.4864 |
| Caraway vs. Fennel | <0.0001 |
| Celery vs. Dill | 0.0006 |
| Celery vs. Fennel | <0.0001 |
| Dill vs. Fennel | <0.0001 |
| ANOVA table | SS | DF | MS | F (DFn, DFd) | P value |
| --- | --- | --- | --- | --- | --- |
| Treatment (between columns) | 1246603 | 7 | 178086 | F (7, 16) = 101.1 | P<0.0001 |
| Residual (within columns) | 28193 | 16 | 1762 | | |
| Total | 1274796 | 23 | | | |

## Slide 21
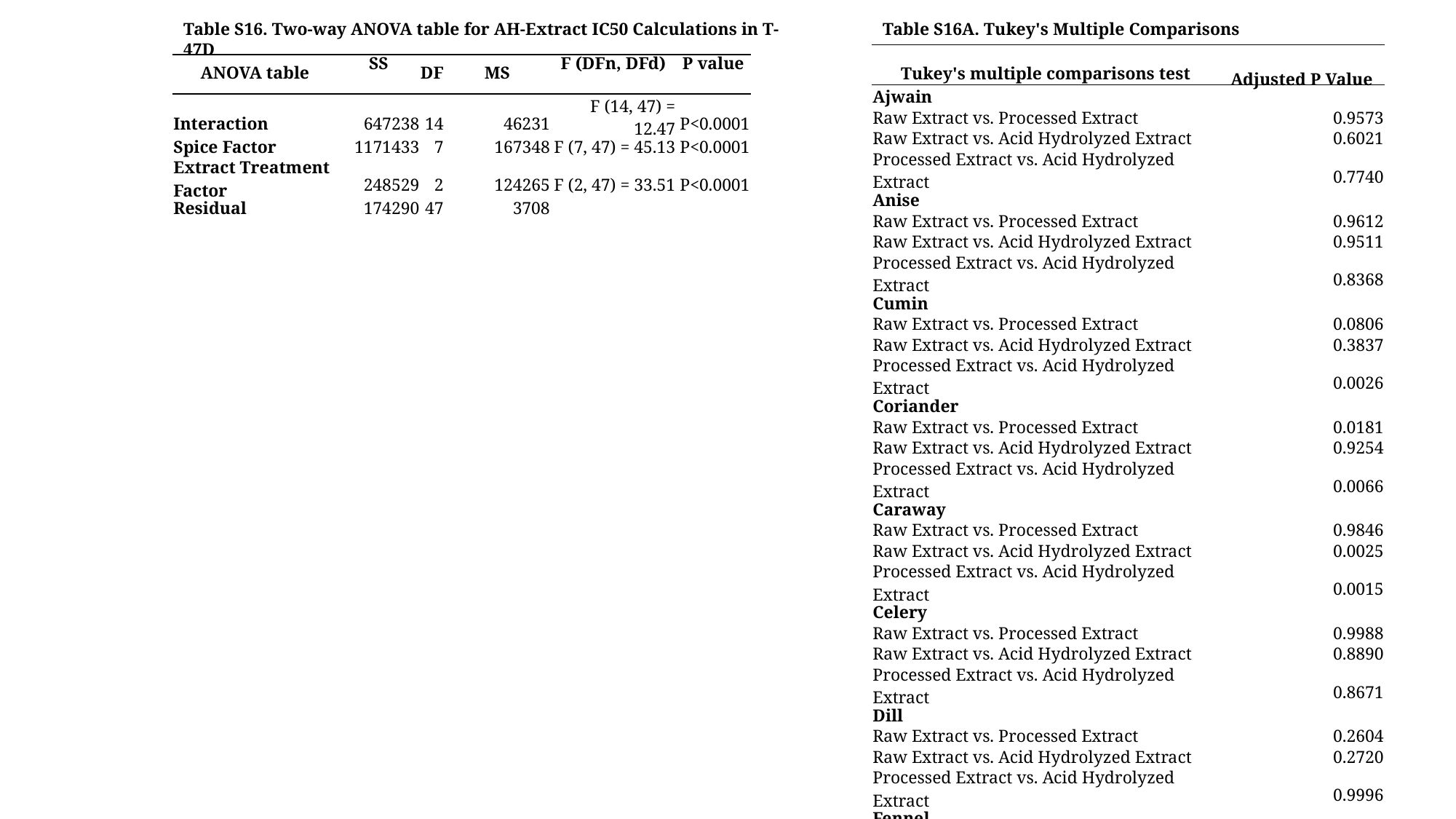

Table S16. Two-way ANOVA table for AH-Extract IC50 Calculations in T-47D
Table S16A. Tukey's Multiple Comparisons
| Tukey's multiple comparisons test | Adjusted P Value |
| --- | --- |
| Ajwain | |
| Raw Extract vs. Processed Extract | 0.9573 |
| Raw Extract vs. Acid Hydrolyzed Extract | 0.6021 |
| Processed Extract vs. Acid Hydrolyzed Extract | 0.7740 |
| Anise | |
| Raw Extract vs. Processed Extract | 0.9612 |
| Raw Extract vs. Acid Hydrolyzed Extract | 0.9511 |
| Processed Extract vs. Acid Hydrolyzed Extract | 0.8368 |
| Cumin | |
| Raw Extract vs. Processed Extract | 0.0806 |
| Raw Extract vs. Acid Hydrolyzed Extract | 0.3837 |
| Processed Extract vs. Acid Hydrolyzed Extract | 0.0026 |
| Coriander | |
| Raw Extract vs. Processed Extract | 0.0181 |
| Raw Extract vs. Acid Hydrolyzed Extract | 0.9254 |
| Processed Extract vs. Acid Hydrolyzed Extract | 0.0066 |
| Caraway | |
| Raw Extract vs. Processed Extract | 0.9846 |
| Raw Extract vs. Acid Hydrolyzed Extract | 0.0025 |
| Processed Extract vs. Acid Hydrolyzed Extract | 0.0015 |
| Celery | |
| Raw Extract vs. Processed Extract | 0.9988 |
| Raw Extract vs. Acid Hydrolyzed Extract | 0.8890 |
| Processed Extract vs. Acid Hydrolyzed Extract | 0.8671 |
| Dill | |
| Raw Extract vs. Processed Extract | 0.2604 |
| Raw Extract vs. Acid Hydrolyzed Extract | 0.2720 |
| Processed Extract vs. Acid Hydrolyzed Extract | 0.9996 |
| Fennel | |
| Raw Extract vs. Processed Extract | 0.0020 |
| Raw Extract vs. Acid Hydrolyzed Extract | <0.0001 |
| Processed Extract vs. Acid Hydrolyzed Extract | <0.0001 |
| ANOVA table | SS | DF | MS | F (DFn, DFd) | P value |
| --- | --- | --- | --- | --- | --- |
| Interaction | 647238 | 14 | 46231 | F (14, 47) = 12.47 | P<0.0001 |
| Spice Factor | 1171433 | 7 | 167348 | F (7, 47) = 45.13 | P<0.0001 |
| Extract Treatment Factor | 248529 | 2 | 124265 | F (2, 47) = 33.51 | P<0.0001 |
| Residual | 174290 | 47 | 3708 | | |

## Slide 22
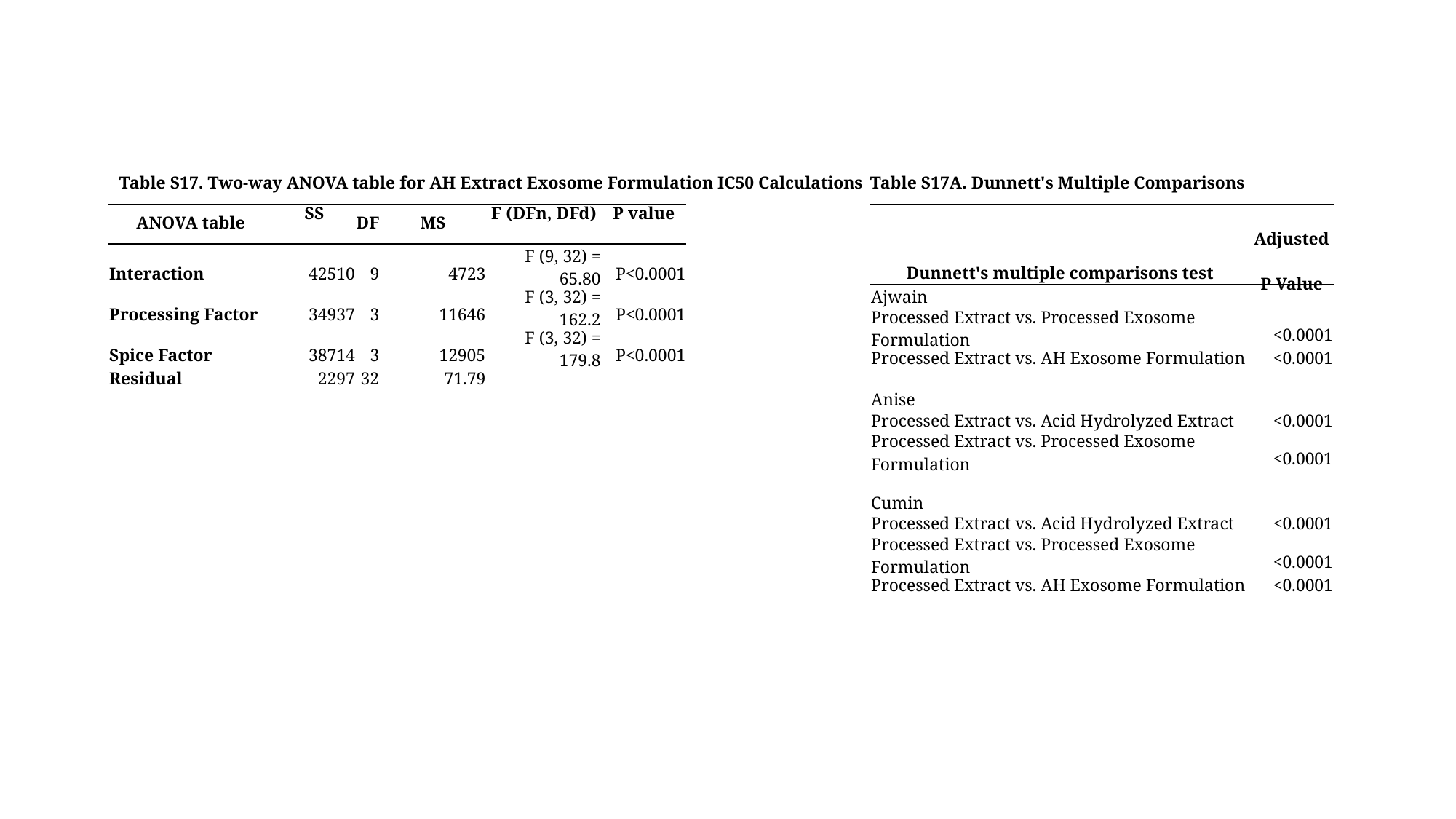

Table S17. Two-way ANOVA table for AH Extract Exosome Formulation IC50 Calculations
Table S17A. Dunnett's Multiple Comparisons
| ANOVA table | SS | DF | MS | F (DFn, DFd) | P value |
| --- | --- | --- | --- | --- | --- |
| Interaction | 42510 | 9 | 4723 | F (9, 32) = 65.80 | P<0.0001 |
| Processing Factor | 34937 | 3 | 11646 | F (3, 32) = 162.2 | P<0.0001 |
| Spice Factor | 38714 | 3 | 12905 | F (3, 32) = 179.8 | P<0.0001 |
| Residual | 2297 | 32 | 71.79 | | |
| Dunnett's multiple comparisons test | Adjusted P Value |
| --- | --- |
| Ajwain | |
| Processed Extract vs. Processed Exosome Formulation | <0.0001 |
| Processed Extract vs. AH Exosome Formulation | <0.0001 |
| | |
| Anise | |
| Processed Extract vs. Acid Hydrolyzed Extract | <0.0001 |
| Processed Extract vs. Processed Exosome Formulation | <0.0001 |
| | |
| Cumin | |
| Processed Extract vs. Acid Hydrolyzed Extract | <0.0001 |
| Processed Extract vs. Processed Exosome Formulation | <0.0001 |
| Processed Extract vs. AH Exosome Formulation | <0.0001 |

## Slide 23
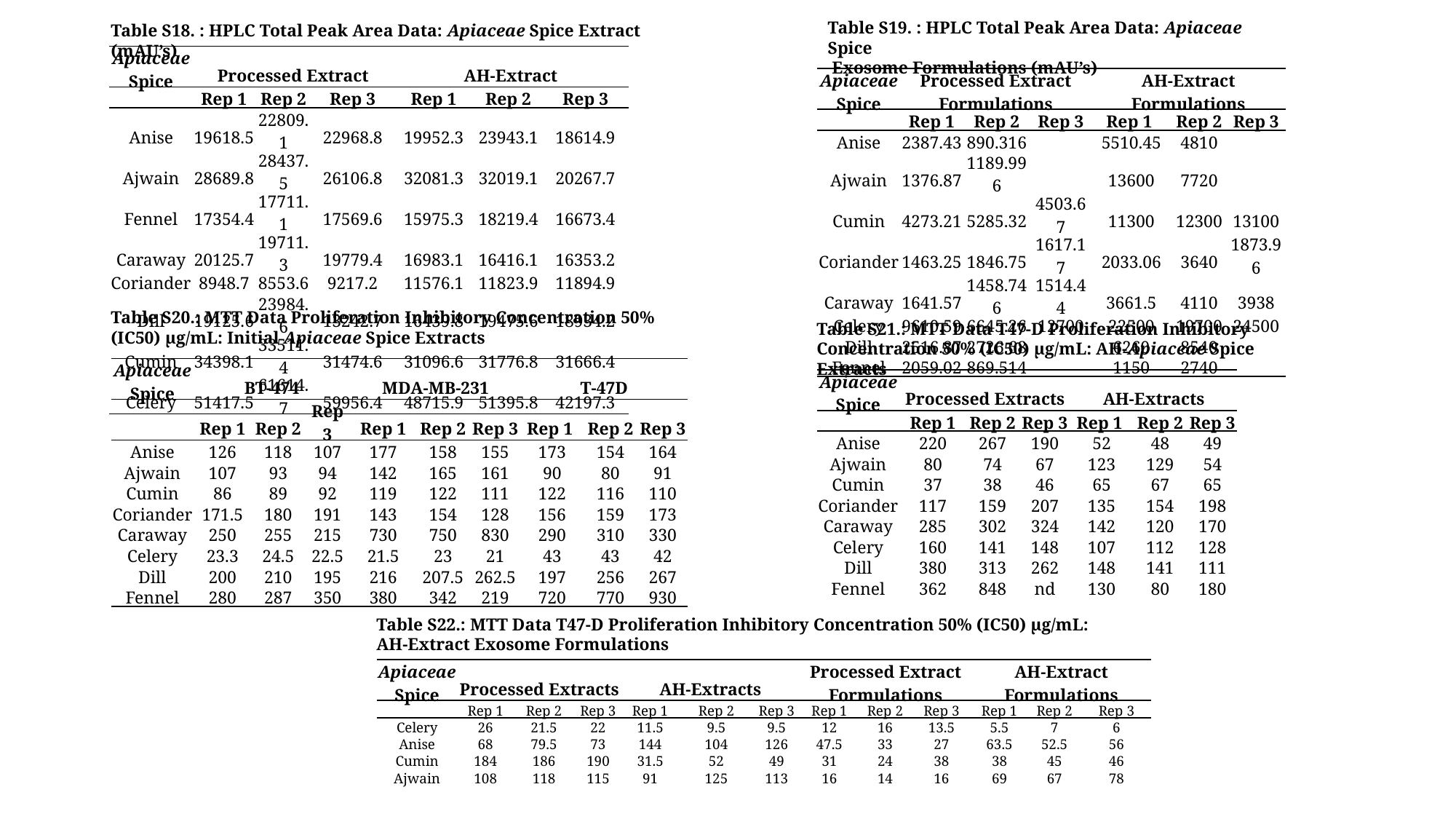

Table S19. : HPLC Total Peak Area Data: Apiaceae Spice
 Exosome Formulations (mAU’s)
Table S18. : HPLC Total Peak Area Data: Apiaceae Spice Extract (mAU’s)
| Apiaceae Spice | Processed Extract | | | AH-Extract | | |
| --- | --- | --- | --- | --- | --- | --- |
| | Rep 1 | Rep 2 | Rep 3 | Rep 1 | Rep 2 | Rep 3 |
| Anise | 19618.5 | 22809.1 | 22968.8 | 19952.3 | 23943.1 | 18614.9 |
| Ajwain | 28689.8 | 28437.5 | 26106.8 | 32081.3 | 32019.1 | 20267.7 |
| Fennel | 17354.4 | 17711.1 | 17569.6 | 15975.3 | 18219.4 | 16673.4 |
| Caraway | 20125.7 | 19711.3 | 19779.4 | 16983.1 | 16416.1 | 16353.2 |
| Coriander | 8948.7 | 8553.6 | 9217.2 | 11576.1 | 11823.9 | 11894.9 |
| Dill | 19125.6 | 23984.6 | 13242.7 | 16439.8 | 19475.6 | 18934.2 |
| Cumin | 34398.1 | 33511.4 | 31474.6 | 31096.6 | 31776.8 | 31666.4 |
| Celery | 51417.5 | 61614.7 | 59956.4 | 48715.9 | 51395.8 | 42197.3 |
| Apiaceae Spice | Processed Extract Formulations | | | AH-Extract Formulations | | |
| --- | --- | --- | --- | --- | --- | --- |
| | Rep 1 | Rep 2 | Rep 3 | Rep 1 | Rep 2 | Rep 3 |
| Anise | 2387.43 | 890.316 | | 5510.45 | 4810 | |
| Ajwain | 1376.87 | 1189.996 | | 13600 | 7720 | |
| Cumin | 4273.21 | 5285.32 | 4503.67 | 11300 | 12300 | 13100 |
| Coriander | 1463.25 | 1846.75 | 1617.17 | 2033.06 | 3640 | 1873.96 |
| Caraway | 1641.57 | 1458.746 | 1514.44 | 3661.5 | 4110 | 3938 |
| Celery | 9610.59 | 6645.26 | 12700 | 22500 | 19700 | 24500 |
| Dill | 2516.87 | 2726.68 | | 6260 | 8540 | |
| Fennel | 2059.02 | 869.514 | | 1150 | 2740 | |
Table S20.: MTT Data Proliferation Inhibitory Concentration 50% (IC50) µg/mL: Initial Apiaceae Spice Extracts
Table S21.: MTT Data T47-D Proliferation Inhibitory Concentration 50% (IC50) µg/mL: AH-Apiaceae Spice Extracts
| Apiaceae Spice | BT-474 | | | MDA-MB-231 | | | T-47D | | |
| --- | --- | --- | --- | --- | --- | --- | --- | --- | --- |
| | Rep 1 | Rep 2 | Rep 3 | Rep 1 | Rep 2 | Rep 3 | Rep 1 | Rep 2 | Rep 3 |
| Anise | 126 | 118 | 107 | 177 | 158 | 155 | 173 | 154 | 164 |
| Ajwain | 107 | 93 | 94 | 142 | 165 | 161 | 90 | 80 | 91 |
| Cumin | 86 | 89 | 92 | 119 | 122 | 111 | 122 | 116 | 110 |
| Coriander | 171.5 | 180 | 191 | 143 | 154 | 128 | 156 | 159 | 173 |
| Caraway | 250 | 255 | 215 | 730 | 750 | 830 | 290 | 310 | 330 |
| Celery | 23.3 | 24.5 | 22.5 | 21.5 | 23 | 21 | 43 | 43 | 42 |
| Dill | 200 | 210 | 195 | 216 | 207.5 | 262.5 | 197 | 256 | 267 |
| Fennel | 280 | 287 | 350 | 380 | 342 | 219 | 720 | 770 | 930 |
| Apiaceae Spice | Processed Extracts | | | AH-Extracts | | |
| --- | --- | --- | --- | --- | --- | --- |
| | Rep 1 | Rep 2 | Rep 3 | Rep 1 | Rep 2 | Rep 3 |
| Anise | 220 | 267 | 190 | 52 | 48 | 49 |
| Ajwain | 80 | 74 | 67 | 123 | 129 | 54 |
| Cumin | 37 | 38 | 46 | 65 | 67 | 65 |
| Coriander | 117 | 159 | 207 | 135 | 154 | 198 |
| Caraway | 285 | 302 | 324 | 142 | 120 | 170 |
| Celery | 160 | 141 | 148 | 107 | 112 | 128 |
| Dill | 380 | 313 | 262 | 148 | 141 | 111 |
| Fennel | 362 | 848 | nd | 130 | 80 | 180 |
Table S22.: MTT Data T47-D Proliferation Inhibitory Concentration 50% (IC50) µg/mL:
AH-Extract Exosome Formulations
| Apiaceae Spice | Processed Extracts | | | AH-Extracts | | | Processed Extract Formulations | | | AH-Extract Formulations | | |
| --- | --- | --- | --- | --- | --- | --- | --- | --- | --- | --- | --- | --- |
| | Rep 1 | Rep 2 | Rep 3 | Rep 1 | Rep 2 | Rep 3 | Rep 1 | Rep 2 | Rep 3 | Rep 1 | Rep 2 | Rep 3 |
| Celery | 26 | 21.5 | 22 | 11.5 | 9.5 | 9.5 | 12 | 16 | 13.5 | 5.5 | 7 | 6 |
| Anise | 68 | 79.5 | 73 | 144 | 104 | 126 | 47.5 | 33 | 27 | 63.5 | 52.5 | 56 |
| Cumin | 184 | 186 | 190 | 31.5 | 52 | 49 | 31 | 24 | 38 | 38 | 45 | 46 |
| Ajwain | 108 | 118 | 115 | 91 | 125 | 113 | 16 | 14 | 16 | 69 | 67 | 78 |

## Slide 24
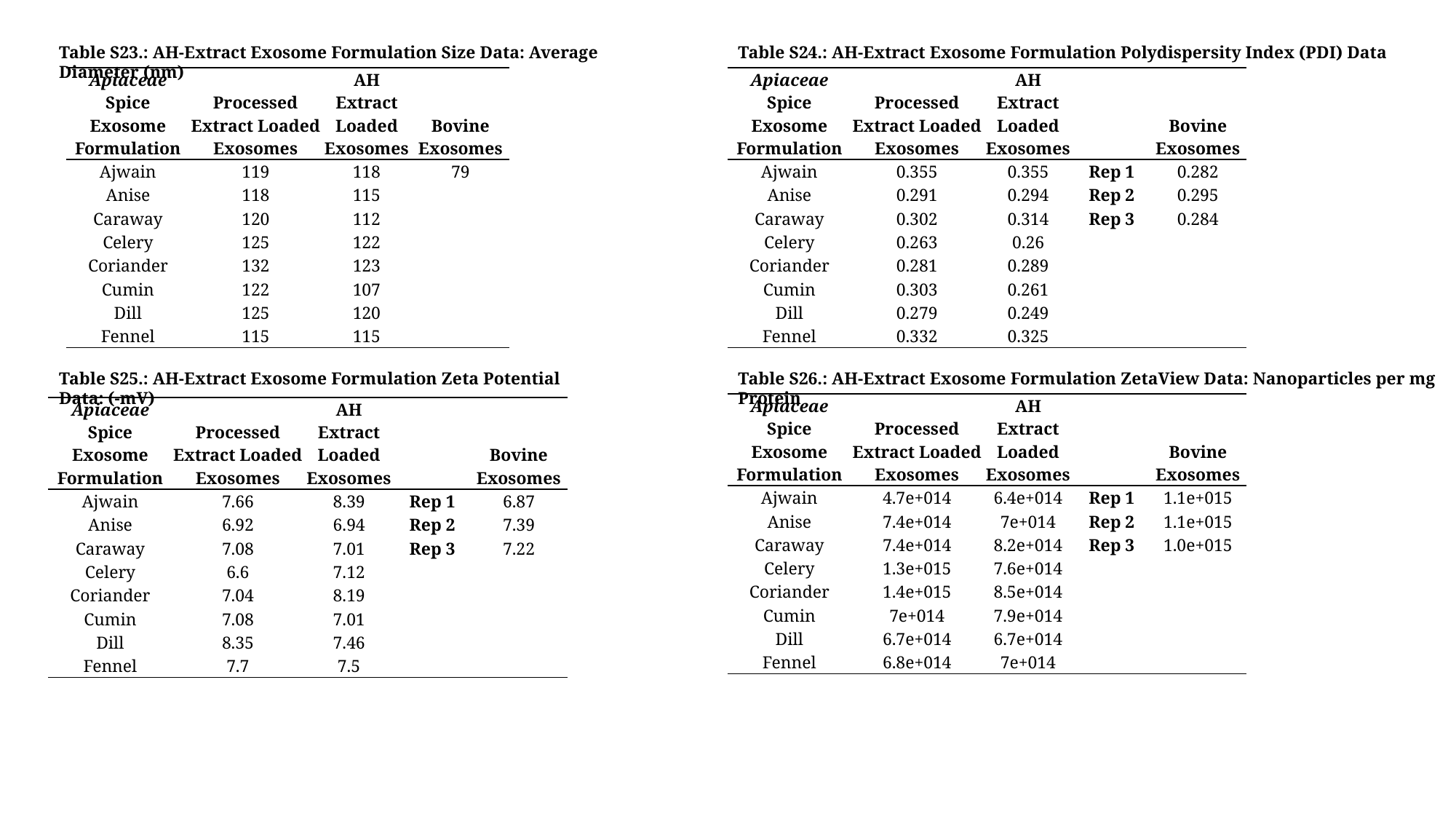

Table S23.: AH-Extract Exosome Formulation Size Data: Average Diameter (nm)
Table S24.: AH-Extract Exosome Formulation Polydispersity Index (PDI) Data
| Apiaceae Spice Exosome Formulation | Processed Extract Loaded Exosomes | AH Extract Loaded Exosomes | Bovine Exosomes |
| --- | --- | --- | --- |
| Ajwain | 119 | 118 | 79 |
| Anise | 118 | 115 | |
| Caraway | 120 | 112 | |
| Celery | 125 | 122 | |
| Coriander | 132 | 123 | |
| Cumin | 122 | 107 | |
| Dill | 125 | 120 | |
| Fennel | 115 | 115 | |
| Apiaceae Spice Exosome Formulation | Processed Extract Loaded Exosomes | AH Extract Loaded Exosomes | | Bovine Exosomes |
| --- | --- | --- | --- | --- |
| Ajwain | 0.355 | 0.355 | Rep 1 | 0.282 |
| Anise | 0.291 | 0.294 | Rep 2 | 0.295 |
| Caraway | 0.302 | 0.314 | Rep 3 | 0.284 |
| Celery | 0.263 | 0.26 | | |
| Coriander | 0.281 | 0.289 | | |
| Cumin | 0.303 | 0.261 | | |
| Dill | 0.279 | 0.249 | | |
| Fennel | 0.332 | 0.325 | | |
Table S25.: AH-Extract Exosome Formulation Zeta Potential Data: (-mV)
Table S26.: AH-Extract Exosome Formulation ZetaView Data: Nanoparticles per mg Protein
| Apiaceae Spice Exosome Formulation | Processed Extract Loaded Exosomes | AH Extract Loaded Exosomes | | Bovine Exosomes |
| --- | --- | --- | --- | --- |
| Ajwain | 4.7e+014 | 6.4e+014 | Rep 1 | 1.1e+015 |
| Anise | 7.4e+014 | 7e+014 | Rep 2 | 1.1e+015 |
| Caraway | 7.4e+014 | 8.2e+014 | Rep 3 | 1.0e+015 |
| Celery | 1.3e+015 | 7.6e+014 | | |
| Coriander | 1.4e+015 | 8.5e+014 | | |
| Cumin | 7e+014 | 7.9e+014 | | |
| Dill | 6.7e+014 | 6.7e+014 | | |
| Fennel | 6.8e+014 | 7e+014 | | |
| Apiaceae Spice Exosome Formulation | Processed Extract Loaded Exosomes | AH Extract Loaded Exosomes | | Bovine Exosomes |
| --- | --- | --- | --- | --- |
| Ajwain | 7.66 | 8.39 | Rep 1 | 6.87 |
| Anise | 6.92 | 6.94 | Rep 2 | 7.39 |
| Caraway | 7.08 | 7.01 | Rep 3 | 7.22 |
| Celery | 6.6 | 7.12 | | |
| Coriander | 7.04 | 8.19 | | |
| Cumin | 7.08 | 7.01 | | |
| Dill | 8.35 | 7.46 | | |
| Fennel | 7.7 | 7.5 | | |
